# Impact of structural modifications of IgG antibodies on effector functions

**DOI:** 10.3389/fimmu.2023.1304365

**Published:** 2024-01-08

**Authors:** Timon Damelang, Maximilian Brinkhaus, Thijs L. J. van Osch, Janine Schuurman, Aran F. Labrijn, Theo Rispens, Gestur Vidarsson

**Affiliations:** ^1^ Sanquin Research, Department of Experimental Immunohematology and Landsteiner Laboratory, Amsterdam, Netherlands; ^2^ Sanquin Research, Department of Immunopathology, Amsterdam, Netherlands; ^3^ Department of Biomolecular Mass Spectrometry and Proteomics, Utrecht Institute for Pharmaceutical Sciences and Bijvoet Center for Biomolecular Research, Utrecht University, Utrecht, Netherlands; ^4^ Department of Antibody Research & Technologies’, Genmab, Utrecht, Netherlands

**Keywords:** antibodies, IgG, subclasses, allotypes, glycosylation, FcγR, complement

## Abstract

Immunoglobulin G (IgG) antibodies are a critical component of the adaptive immune system, binding to and neutralizing pathogens and other foreign substances. Recent advances in molecular antibody biology and structural protein engineering enabled the modification of IgG antibodies to enhance their therapeutic potential. This review summarizes recent progress in both natural and engineered structural modifications of IgG antibodies, including allotypic variation, glycosylation, Fc engineering, and Fc gamma receptor binding optimization. We discuss the functional consequences of these modifications to highlight their potential for therapeutical applications.

## Introduction

Antibodies, also known as immunoglobulins (Ig), are among the most abundant protein components in the human blood, constituting about 20% of the total protein in plasma by weight. The five major classes of Ig in humans are IgG (70-85%), followed by IgA (5-15%), then IgM (5-10%), with trace amounts of IgD (~0.25%) and IgE (<0.25% of the total serum Igs) (1, 2). These glycoproteins share similar structures and composition (82–96% protein and 4–18% carbohydrate), but differ in size, charge, amino acid sequence and effector function. The most abundant isotype in healthy human serum, IgG, can further be divided into four subclasses: IgG1 (60–70% in plasma), IgG2 (20–30%), IgG3 (5–8%) and IgG4 (1–3%).

**Figure 1:**
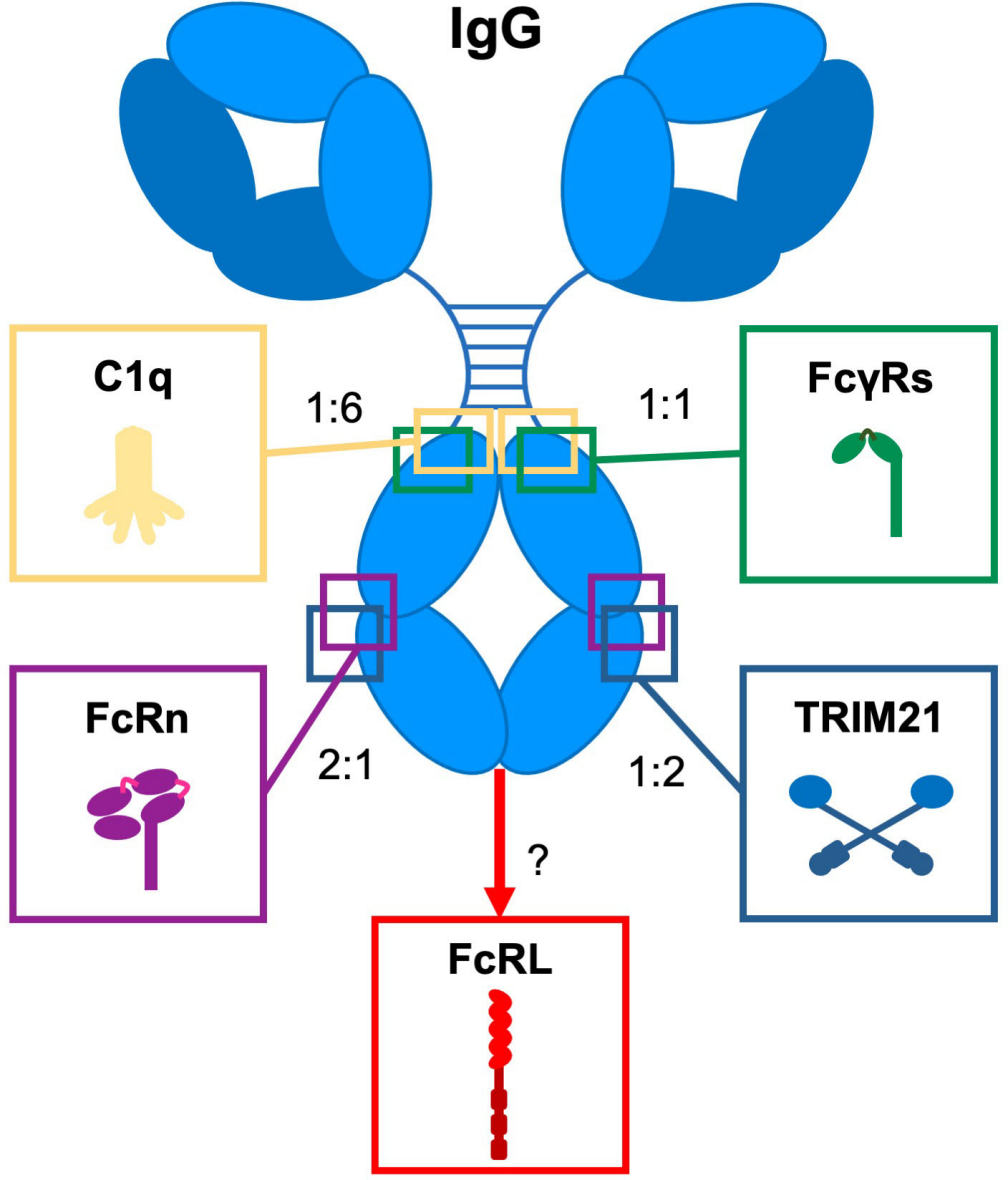
Interaction of IgG with Fc effector molecules. Schematic representation of IgG and its Fc-engaging molecules (complement component (C1q), Fc gamma receptors (FcγRs), the neonatal Fc receptor (FcRn), Tripartite motif 21 (TRIM21), and Fc receptor-like (FcRL) molecules through which antibodies exert their biological activity. For each ligand, the binding site on IgG and the stoichiometry of the interaction with IgG is indicated. Adapted from (3).

IgG antibodies are major effector molecules of the humoral immune system. They provide a link between the adaptive immune system and the effector mechanisms of the innate immune system through high affinity antigen-specific recognition of foreign structures. This can result in either simply blocking the interactions of the foreign molecules with their ligands or in the formation of high avidity interactions with innate molecules, such as complement, and effector cells through Fc gamma receptors (FcγRs), and the neonatal Fc receptor (FcRn) (**Figure 1**) inducing effector functions (4). Variation in the IgG subclass structures shapes the biological effector activities, in broad terms, IgG1 and IgG3 are more potent in inducing these effector functions, while IgG2 and IgG4 do less so.

Given the extensive scope of the research on IgG antibody modifications, this review focuses only on the major natural and engineered changes, and provides literature references for further reading on variants not covered. We will discuss how they affect structure and functional modalities of IgG themselves and binding to various IgG receptors, introduced below.

### Complement

Both IgM and IgG target-bound antibodies can activate the classical complement pathway via the initial binding of hexameric C1q. Of the four IgG subclasses, IgG1 and IgG3 are the most efficient activators of the classical complement pathway; IgG2 and IgG4 (serine in position 331 severely reduces complement binding) require high antigen densities or repeated polysaccharide structures (5, 6). However, given that monomeric IgG has a low binding affinity to the individual globular heads of C1q, it requires multimerization to form a polyvalent, ideally hexameric, high avidity interaction platform for C1q (7–11). The process of multimerization (or hexamerization) is highly dependent on the size of the antigen, expression level, spatial distribution and mobility but also the epitope position, binding angle, hinge length and flexibility (10, 12). Especially the hinge length can influence how and where complement fragments of C4 are deposited (13). These distinctions will be discussed in more detail below.

### FcγRs

Humans express five FcγRs: FcγRI, FcγRIIa, FcγRIIb/c, FcγRIIIa, and FcγRIIIb. IgG1 and IgG3 bind with higher affinity than IgG2 and IgG4 to FcγRs on effector cells (**Table 1**). However, the structural determinants responsible for the subclass-specific affinity variation are still largely unknown, except for binding to FcγRI, which is reduced for IgG4 due to the presence of S331 and F234 in comparison to P331 and L234 in IgG3 (14) and IgG1 (15). In addition, monomeric IgG2 Abs only bind FcγRIIa, most likely due to their short hinges lacking G236 (16). It is important to note that complexed IgG2 was found to bind FcγRIIIa 158V, presumably due to multivalency-induced avidity effects, that might be relevant in (auto) immune responses (17). The interaction of IgG antibodies with FcγRs on effector cells has been recognized as one of the most critical immune response determinants against infections (18–23). Induced effector functions are also influenced by natural (allotypes) and engineered amino acid changes in the Fc region of IgG antibodies, glycosylation profiles, FcγR polymorphisms as well as FcγR expression profiles on different immune cells.

**Table 1:** Properties of human IgG subclasses.

Structure	IgG1	IgG2	IgG3	IgG4
H chain type	γ1	γ2	γ3	γ4
Molecular mass (kDa)	146	146	170	146
Amino acids in hinge region	15	12	47-62†	12
Disulfide bonds in hinge region	2	4	11	2
Ig H constant gene	IGHG1	IGHG2	IGHG3	IGHG4
Alleles of IGHG	15	18	29	8
*N*-linked glycans per H chain	1	1	2**†**	1
O-linked glycans per H chain	0	0	3	0
Biology				
Mean adult serum level (g/L)	6.98	3.8	0.51	0.56
Proportion of total IgG (%)	43-75	16-48	1.7-7.5	0.8-11.7
Half-life (days)	21	21	7/~21**†**	21
Placental transfer	+++	++	++/+++**†**	++
Ab targets				
Proteins	++	+/-	++	++*
Polysaccharides	+	++	+	+
Allergens	+	(-)	(-)	++*
Binding capacity				
Complement (C1q)	++	+	+++	+/-
FcγRI	+++	-	+++	++
FcγRIIa	++	(+)/++**‡**	++	+
FcγRIIb/c	+	+/-	++	++
FcγRIIIa	++**‡**/+++**§**	+/-**§**	++**†‡**/+++**†**	-/++**§**
FcγRIIIb	+**‡§**	+**§**	+**‡§**	+/-**§**
FcRn (at pH <6.5)	+++	+++	++/+++**†**	+++
FcRL4	-	-	+	+
FcRL5	++	+	+	++
TRIM21	+++	+++	+++	+++
Binding to Protein A	+++	+++	-/+++**†**	+++
Binding to Protein G	+++	+++	+++	+++
Effector functions				
Complement (CDC)	++	+**†** ***#***	+++	-
Phagocytosis (ADCP)	+++	+**‡**	+++	+
Cytotoxicity (ADCC)	+++	+**†‡**	+++**†**	+

**†** IgG allotype-specific

**‡** FcγR allotype-specific

* after repeated exposure

**§** depending on IgG fucosylation status

**#** against repeated/polysaccharide structures

### FcRn

IgG antibodies also bind FcRn, which mediates their half-life, placental transport, and bidirectional transportation to mucosal surfaces (24, 25) and orchestrates cellular responses to immune complexes (26–29). The interaction with FcRn is highly pH dependent and only occurs at acidic pH (pH < 6.5) as present in the endosomes. After recycling or transport to the cell surface, the IgG is released again at physiological pH, as present outside of the cells. Naturally occurring or bio-engineered mutations can impact the interaction with FcRn.

### Alternative receptors

IgG also bind less studied and underappreciated effector molecules, including the two members of the FcR-like (FcRL) family (FcRL4 and FcRL5) (30, 31), and tripartite motif-containing protein 21 (TRIM21) (32, 33). Although controversial, DC-SIGN has been described to bind to sialylated IgG (34, 35). However, more recent studies found no detectable binding of human IgG Fc to DC-SIGN, indicating that DC-SIGN might not be an IgG receptor after all, at least not in humans (36–38).

FcRL molecules, which are part of the Ig superfamily and mainly expressed on B cells, interact with IgG antibodies (39). FcRL4 only binds IgG3 and IgG4, while FcRL5 is able to bind all IgG subclasses (30, 40). The exact binding epitope of FcRL5 on IgG has not been described yet, but a complex binding interaction was hypothesized, in which both the Fab- and Fc-fragments of IgG are involved (40).

Human IgG also binds to TRIM21, an intracellular cytosolic IgG receptor and E3-ligase, with nanomolar affinity through its PRYSPRY domain, which is highly conserved (41, 42). TRIM21 is relevant in the context of antibody-dependent intracellular neutralization of viruses and transcriptional activation of several immune regulator genes (33). The neutralization itself activates proteasome-dependent degradation of the antibody based targets that also seem to be activated in mouse tau immunotherapy models (43).

Here we discuss natural and engineered structural modifications of IgG antibodies that can impact the interactions with complement, FcγRs, FcRn and resulting downstream effector functions.

## Structure of IgG antibodies

**Figure 2:**
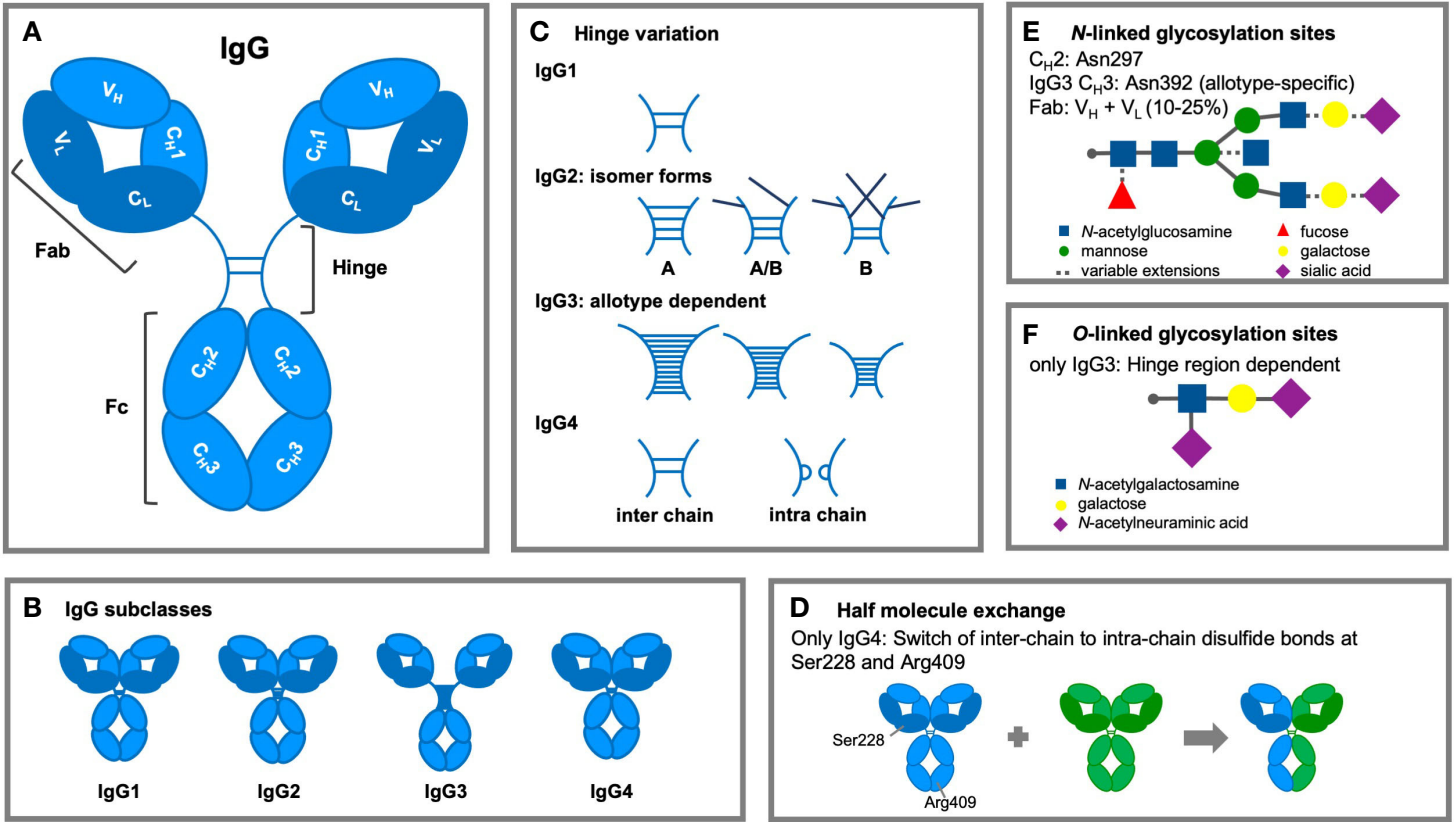
Structural modifications of IgG antibodies. **(A)** IgG1-4 antibodies consist of four polypeptide chains, composed of two identical heavy (H; light blue) chains of 50 kDa and two identical light (L; dark blue) chains of 25 KDa, linked together by interchain disulfide bonds. Each H chain consists of an N-terminal variable domain (VH) and three constant domains (CH1, CH2, and CH3). IgG molecules are joined by a flexible stretch of polypeptide chain between CH1 and CH2, known as the hinge region. The VH and CH1 domains and the L chains form the fragment antigen binding (Fab) region. The lower hinge region and the CH2/CH3 domains form the fragment crystalline (Fc) region, which interacts with effector molecules and cells. **(B)** The length (IgG1: 15 amino acid residues, IgG2: 12 residues, IgG3: 32-64 residues, and IgG4: 12 residues) and flexibility of the hinge region varies among the IgG subclasses. **(C)** Subclass differences in hinge flexibility are also impacted by differential number of inter-chain disulfide bonds (IgG3: 11 bonds; IgG1 & IgG4: two bonds; IgG2: four bonds), both IgG2 and IgG4 are found as several isomers. Darker disulfide bonds indicate that they are linked to the light chain due to light chain reshuffling of the C-C bonds. **(D)** IgG4 antibodies can split into two half-molecules (one H chain + one L chain) that can then randomly form complete monovalent-bispecific Abs, which is either termed half molecule exchange or Fab arm exchanged. **(E)** Within the CH2 region is one N-linked glycosylation site containing carbohydrate groups attached to asparagine 297. The highly conserved glycan has a heptasaccharide core and variable extensions, such as fucose, galactose and/or sialic acid (dashed line). Additional N-linked sites have been reported in the antigen-binding region and allotypic variants of IgG3 at position asparagine 392. **(F)** The hinge region of IgG3 exhibits O-linked glycosylation sites.

IgG antibodies are composed of two distinct fragments, the antigen-binding Fab and the Fc domain, which interacts with effector molecules of the immune system (**Figure 2A**). IgG molecules are generally composed of two identical γ H polypeptide chains of 50 kDa and two identical κ or λ L polypeptide chains of 25 kDa, linked via inter-chain disulfide bonds. Each H chain consists of an N-terminal variable domain (V_H_) and three constant domains (C_H_1, C_H_2, C_H_3) connected by a flexible stretch of polypeptide chain, known as the hinge region. The encoding human Ig H constant (IGHC) genes are *IGHG3*, *IGHG1*, *IGHG2*, and *IGHG4*, from 5’ to 3’ in the IGH locus on chromosome 14q32.33 (44). The L chains also consist of an N-terminal variable domain (V_L_) and a constant domain (C_L_). In association with the V_H_ and C_H_1 domains, the L chains form the Fab regions, allowing the V regions to shape the antigen-binding region (45). Rearrangement of gene segments and somatic mutations generate variations in the amino acid sequence of the N-terminal domains. This results in variable regions, containing six hypervariable loops, known as complementarity determining regions (CDRs) (46), which together form the antigen recognition site (paratrope) in the Fab region. The lower hinge region and the C_H_2/C_H_3 domains form the Fc region, which interacts with effector molecules and immune cells. The four IgG subclasses show a homology of more than 90% on the amino acid sequence level of their “constant” domains, but are highly divergent in the hinges and upper C_H_2 domains. These regions are crucial for binding to FcγR (mainly the residues L234, L235, D265 and S298) and C1q, influencing various effector functions such as phagocytosis, antibody-dependent cell-mediated cytotoxicity (ADCC), and complement activation.

### Structural variation in the hinge region

The length and flexibility of the hinge region varies among the IgG subclasses (**Figure 2B**) (47). This affects the conformations of the Fab arms relative to the Fc domain as well as to each other and allows the Fab arms to bind to multiple targets and the Fc to interact independently with effector molecules of the immune system. There are ongoing efforts to determine the diversity of conformational structures and flexibility of the IgG molecules with particle electron tomography (48). This flexibility is strongly affected by the hinge length which varies considerably between the subclasses (IgG1: 15 amino acid residues, IgG2: 12 residues, IgG3: 32-64 residues, and IgG4: 12 residues; **Table 1**).

The lower hinge region of IgG2 (encoded by the C_H_2 exon) lacks one of the double glycines found at position 236-237. This and up to four inter-heavy chain disulfide bridges restrict the flexibility of the IgG2 molecule (16, 49, 50). Similarly, the shorter hinge of IgG4 gives it less flexibility than the one of IgG1 (51). IgG3 has a much longer hinge region than any other IgG subclass (containing up to 62 amino acid residues), but its length is allotype-specific (discussed below). Recently, Bashirova et al. detected a single individual carrying an allele with five *IGHG3* hinge exon (77 amino acid residues) (52).

An IgG3 hinge region can consist of up to 2x21 prolines and 11 disulfide bridges, which results in a poly-proline helix with limited flexibility (51). However, the Fab fragments in an IgG3 molecule are relatively far away from the Fc fragment, giving the entire molecule a greater reach with consequences for downstream effector function, such as ADCC and complement activation (3, 53). The relative flexibility of the Fab arms and the Fc differs following the same order of the hinge length: lgG3 > lgG1 > lgG4 > lgG2 (47).

### Inter-chain disulfide bonds

Another structural difference between the human IgG subclasses is the linkage of the H and L chain by disulfide bonds. This bond links the carboxy-terminal cysteine of the L chain to the cysteine at position 220 (in IgG1) or at position 131 (in IgG2, IgG3, and IgG4) in the C_H_1 domain. A pair of cysteines in close proximity will form a disulfide bond that fixes and stabilizes the tertiary structure of an IgG molecule, which is essential for the function of the molecule (54). Besides subclass differences in hinge flexibility due to differential number of inter-chain disulfide bonds (IgG3: 11 bonds; IgG1 & IgG4: two bonds; IgG2: four bonds), both IgG2 and IgG4 are found as several isomers, in which the hinge disulfide bonds are differentially interconnected (51) (**Figure 2C**).

Three main isomeric variants of the IgG2 hinge have been described (IgG2A, B and A/B) (49, 55). These structural isomers were first found in recombinant monoclonal IgG2, however, were later confirmed to be present in serum from healthy and diseases subjects. Their presence is a consequence of alternative disulfide bond formation between the C-terminal cysteine of the LC and a cysteine in the C_H_1 domain of the heavy chain (55) and was found to be more prevalent in IgG2 with kappa LCs (56). The IgG2A isomer may confer more flexibility to the Fab arms relative to IgG2B, which can have consequences for downstream effector functions, even though FcγR binding does not seem to be different for the two isomers (57). This seems to strongly affect how IgG2 interacts and cross-links its target, which can lead to superagonistic antibodies when targeting cellular antigens. These superagonistic effects are independent of the Fc (58).

The two isomers of IgG4 differ in the disulfide bonding of hinge cysteines that are classically bonding the two H chains. However, these disulfide bonds are in flux, as they are a subject of reduction and re-oxidation, forming intra-chain disulfide bonds between cysteines found at positions 226 and 229, resulting in non-covalently linked half-molecules in addition to covalently linked inter-chain isomers (59, 60).

### IgG4 Fab arm exchange

IgG4 antibodies are unique and dynamic molecules due to their ability to undergo a process called Fab arm exchange (FAE; **Figure 2D**). *In vivo*, an IgG4 antibody can reassemble after secretion, recombining two halves of two random IgG4 (one H chain and one L chain) to form functionally monovalent-bispecific antibodies (59, 60).

Two amino acids seem required for FAE *in vivo.* One is the serine at position 228 in the core hinge region of IgG4, and the other one is arginine at position 409, which results in weaker C_H_3-C_H_3 interactions (60, 61). These two amino acids at positions 228 and 409 are unique to IgG4, which might cause a conformational change that could explain the poor FcγR and C1q binding properties of IgG4 (62). Interestingly, the arginine residue at position 409 is polymorphic as the IGHG4*03 allele harbors a lysine at that position (allotypes discussed below). Nevertheless, this allotype, regardless of containing S228 is not prone to FAE, showing that both serine at position 228 and arginine in position 409 are essential (59).

IgG4 antibodies that underwent FAE cannot effectively crosslink the target antigen. This effect in combination with the requirement for repeated antigen exposure, low affinity to activating FcγRs and complement, may contribute to the anti-inflammatory properties of IgG4 (59, 63). In this way, the strong immune effector functions otherwise provided by IgG1, IgG3 and even IgG2, are likely to be toned down after class switching to IgG4, both by the lesser interactions Fc-receptors and complement, but also due to its monovalency. IgG4 is therefore generally regarded as less important subclass in the autoimmune setting. However, IgG4 can still be highly pathogenic, for example in the form of (blocking) autoantibodies in pemphigus (64), primary membranous nephropathy (65, 66), or in IgG4-related disease (67). Elevated levels of serum IgG4 have also been associated with asthma (68) and tissue eosinophilia (69). Even more intriguingly, the monovalency of IgG4 may even be required in myasthenia gravis when targeting muscle-specific kinase (MuSK) where monovalency of IgG4 can be required to block neuromuscular signaling and produce pathogenicity (70–72).

## Interindividual variations of IgG antibodies: allotypes

**Figure 3:**
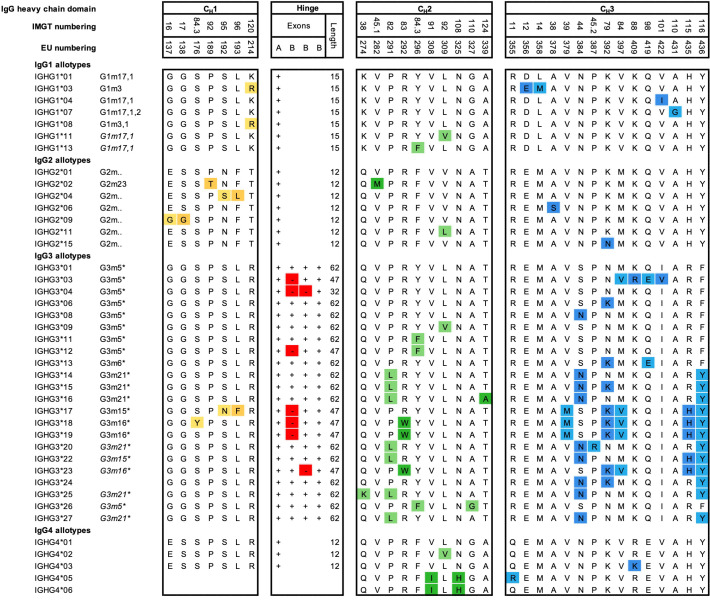
Amino acid variation between IgG allotypes. Variation at the amino acid level between IgG allotypes within the IgG1, IgG2, IgG3, and IgG4 subclasses according to the Reference to ImMunoGeneTics information system (IMGT). For each domain, CH1, hinge, CH2 and CH3, amino acid differences between polymorphic variants are indicated. Polymorphisms in the hinge region are identified by the presence or absence of hinge exons **(A, B)** (3). Additional ‘silent’ mutations exist but are only visible on the genetic level. Historical nomenclature (Gm), based on serology, are included, but indicated in italics for new alleles, that have not been assigned a name to in accordance with Gm system.

Allotypes describe antibody genetic markers (Gm) on the constant regions of antibodies, likely found across all isotypes and light chains, but most studied for the four IgG subclasses (**Figure 3**). A large number of polymorphic IgG variants were originally discovered due to serological reactivities to IgG originating from other individuals (alloreactivity) (73), which were used to study population variations (74). However, sequencing efforts of the IgG genes from different ethnic groups revealed that even more polymorphic variants exist, especially for IgG3 (75, 76). Non-synonymous mutations are the basis for differences in primary amino acid sequences between polymorphic variants. Polymorphic variants have been identified on the γ1-4, α2, and ϵ H chains of the subclasses IgG1-4, IgA2 and IgE, and on the κ L chain (Km allotypes) (77–79). Currently, 27 Gm polymorphisms have been identified and categorized in the ImMunoGeneTics information system (IMGT), which correspond to changes in the amino acid sequence of the constant region (encoded by 15 *IGHG1*, 18 *IGHG2*, 29 *IGHG3*, and 8 *IGHG4*). An overview of all the IgG allotypes, which differ in amino acid sequence, is presented in **Figure 3**. However, numerous additional single mutations do exist on a genetic level, which do not result in amino acid changes and are not shown in the figure (79). Recently, 28 novel variant sites in *IGHG1-3* alleles were discovered in seven genetically isolated Brazilian population (76). In addition, 11 novel alleles and 17 extensions of known IGHG and IGHM alleles were observed in a study using a novel sequencing technique (80). This demonstrates that the diversity of the IGHG genomic region is still far from being fully characterized in whole genome sequencing databases (81).

### Haplotypes and non-coding allotype-associated regions

IgG allotypes were found to correlate with plasma IgG levels (82, 83), which has been hypothesized to be the result of variation in the non-coding switch regions or unfavorable RNA transcripts. IgG allotypes can affect class-switching efficiency and thereby serum isotype and subclass concentrations (83–86). The concentration of IgG1 is lower in individuals with the IGHG1*03 allele in combination with IGHG2*02 and any of IGHG3*01/*04/*06/*09/*11/*12 as compared with those who lack this combination (82). IgG3 polymorphisms within sterile promoter region that undergo transcription preceding class switching could affect both class-switching efficiency directly and antibody serum levels (83, 85). This could explain why higher lgG3 levels were found in individuals carrying IGHG3*01/*04/*06/*09/*11/*12 compared to IGHG3*14/*15/*16 (82).

IgG1 allotypes have also been associated with changes in antibody subclass distribution, magnitude and functionality in specific responses, such as to HIV envelope glycoprotein vaccination (86) or to opsonized herpes simplex virus-infected fibroblasts (87–89). The IGHG2*02 allele containing a V282M substitution in the C_H_2 domain and a P189T substitution in the C_H_1 domain seems to grant a functional advantage against bacterial infections, but the mechanism behind this is not understood (90). IgG3 allotypes with longer half-life (discussed below) and good transplacental transport (R435H) (91, 92), although produced at lower levels (83), may provide better protection against infectious diseases (93). Other reported associations between human IgG allotypes and disease conditions include autoimmunity (94, 95) and cancer (96, 97).

### Functional consequences due to allotypic diversity

IgG3 is the most polymorphic subclass due to various levels of molecular diversity observed with the *IGHG3* alleles (**Figure 3**). IgG3 allotypic variations can have structural and functional consequences, such as shorter hinge regions and extended half-life compared to other allotypes (17, 91). The hinge region is encoded by one A exon, and from one to three 15 amino acid long B exons, depending on the G3m alleles, so it can vary from 32 to 62 amino acids and can influence structural conformations (75). The length of the hinge region can influence the capacity of IgG3 allotypes to induce effector functions, such as ADCC, likely through altered proximity at the immunological synapse (3). It has also been demonstrated that IgG3 allotypes with a phenylalanine at position 296 (IGHG3*11 and IGHG3*12), or a tryptophan at position 292 (IGHG3*18 and IGHG3*19) exhibit a lower affinity to FcγRIIIa and ADCC activity. In addition, IgG3 allotypes with a leucine at position 291 (IGHG3*14, IGHG3*15, and IGHG3*16) also showed reduced ability to mediate ADCC, but without apparent changes in affinity to FcγRIIIa (3). The same study also found that IgG3 antibodies with a short hinge, e.g., IGHG3*04 (2 exons), exhibited the strongest ADCC capacity, but this was not reflected by an increased affinity for the receptor FcγRIIIa (3). This reduced hinge length is similarly associated with increased ADCC against HIV infected cell lines (98), but also increased inflammation and death in cerebral malaria (99). This shorter synapse due to the shorter IgG3 hinge seems to reflect on the capacity of anti-CD20 antibodies to increase ADCC against CD20^+^ tumor cells (100). Curiously, this enhancement of ADCC by shorter distance between effector and target cell was suggested to result in less phagocytosis (98, 100), which may quench antibody inflammatory properties.

The two IgG3 variants IGHG3*01m (GenBank : MK679684) and IGHG3*17, both carry mutations at amino acid positions 419 (glutamic acid) and 392 (lysine) respectively. Although these positions do not define these allotypes, they are implicated in improved binding to FcγRIII and FcγRIIb receptors and enhanced ADCC responses in anti-HIV antibody responses (81).

In addition, polymorphisms in the C_H_3 domain affect the C_H_3-C_H_3 interdomain interactions (60), with potential consequences for both complement activation (60, 101) and aggregation dynamics (102, 103). IgG3 binds with a higher affinity to C1q in comparison to other IgG subclasses (104). While this affinity is believed to be associated with the enhanced flexibility of IgG3 rather than the length of its hinge region (105) under conditions of low antigen density (106). A more recent study showed that antigen density and antibody hinge length play an important role in antibody-mediated CDC. In addition, the study identified that although the differences between IgG1, IgG3 and IgG4 allotypes were minor, the allelic variant IGHG2*06, containing a unique serine at position 378 in the C_H_3 domain, showed less efficient complement activation and CDC compared to other IgG2 polymorphisms (107).

IgG3 has a shorter half-life (~ seven days) than the other three subclasses (21 days). This is because FcRn-mediated transport of IgG3 is inhibited in the presence of IgG1 which seems to be a net result of a less compatible pH-dependent binding of IgG3 containing arginine at position 435, a key-interacting site for FcRn binding (91). However, individuals with certain IgG3 allotypes (**Figure 3**), harbor a histidine at position 435, which makes their IgG3 half-life comparable with that of IgG1 (91). The same rational applies to FcRn placental transport of the different IgG3 variants (92). In addition, amino acid modifications remote from the FcRn binding site can also affect IgG binding to FcRn (108, 109). Specific allotypes also influence the purification of human IgG3 from serum samples, as only allotypes containing a histidine residue at position 435 can be purified by using protein A (110). To purify IgG3 allotypes without a histidine at position 435, protein G can be used (110).

The functional differences of IgG allotypes, including the impact of differences in the hinge domain, on expression levels, half-life, FcγR and FcRn binding (111), complement activation (7, 111, 112), antigen binding, and immunogenicity are still understudied and relatively unknown. Allotypes may also be relevant to understand the pathogenesis of different infectious diseases, and to exploit immune responses to develop novel antibody-based therapeutics or vaccines.

## Glycosylation

Just like most proteins, antibodies can be glycosylated as a post-translational modification. The glycosylation, and differences thereof, in the Fc and Fab domains of the antibody has a critical impact on antibody function e.g., complement activation or ADCC, which will be discussed in the paragraphs below. Antibodies can contain O-linked glycans added to serines or threonines, or *N*-linked glycans added to asparagine (N), if so-called NxS/T-motives are present, which consist of an asparagine, then any amino acid except proline, followed by a serine or threonine. Both O- and *N*-linked glycan additions occur in the lumen of the endoplasmic reticulum or Golgi apparatus (113). The composition of *N*-linked glycans seems to be in part regulated by the type of antigen, a range of B cell stimuli, including stress, disease, cytokine activity, and innate immune signaling receptors (114, 115). Besides V(D)J recombination, somatic hypermutation, and class switch recombination, *N*-linked glycosylation can be considered as an additional mechanism of antibody diversification (116, 117).

### *N*-linked glycosylation of IgG

IgG generally contains a single highly conserved *N*-linked glycan at position 297. The core structure of the IgG *N*-linked glycans comprises *N*-acetylglucosamine (GlcNAc) and mannose residues with possible extensions with galactose, sialic acid, core fucosylation, and bisection of GlcNAc residues (115). Bisection describes an additional GlcNAc branch on the first mannose residue on an *N*-linked glycan (56) (**Figure 2E**). The relative abundance of the different glycoforms for global IgG can be influenced and altered by multiple factors, including age, pregnancy, sex (118), epigenetics (119), disease state (120–122). The glycans at N297 can influence the quaternary structure of the Fc region and therefore the antibody stability (123, 124). It also has a great impact on the ability of antibodies to bind to FcγRs and complement (125, 126), which consequently modulates effector functions, such as ADCC and complement-dependent cytotoxicity (CDC) (22, 127–133). For instance, the removal of glycans at this position (N297) abrogates binding to all FcγRs and C1q except for the high affinity FcγRI which retains minor binding after deglycosylation (134).

The core fucose affects binding to FcγRIIIa/b (127, 135), with non-fucosylated antibodies binding FcγRIII much stronger than fucosylated antibodies. The absence of fucose translates to an up to 20-fold higher FcγRIIIa affinity (127, 136, 137). This effect can be even further increased to ~40-fold for FcγRIIIa by hyper-galactosylation of afucosylated IgG1 (128). These elevated affinities seem to translate to even higher enhancement of Natural Killer (NK) cell-mediated ADCC (127, 128, 130, 138, 139). The importance of afucosylated IgG-induced immune responses in humans has been identified in various diseases (22, 140–146). Additionally, antigen-specific glycosylation can vary significantly depending on the nature of the antigen as seen in some infections (21, 141, 146, 147), and vaccinations (148–150). Despite its relevance, the context in which afucosylated antibody responses are formed, remains understudied.

Recent data also suggests the functional importance of increased Fc galactosylation and sialylation after both infection (146) and vaccination (148, 150, 151). Whereas galactose has a positive effect on IgG binding to FcγRs especially in combination with afucosylation (152), sialylation seems to have either no or minor negative effects on the binding to FcγR (128, 153). However, human IgG antibodies containing terminal sialic acid on their Fc *N*-glycans have been shown to reduce ADCC (154). In addition, studies on intravenous immunoglobulin (IVIg) activity in models of inflammatory arthritis, immune thrombocytopenia and epidermolysis bullosa acquisita showed that cleavage of the terminal sialic acid residues of the Fc fragment of IVIg can reduce its anti-inflammatory activity (155–157). However, whether this effect is also true in humans, and if it is channeled through DC-SIGN and similar receptors remains highly controversial as discussed above (37, 38).

IgG-Fc galactosylation seems to promote C1q binding and complement activity (133, 158), whereas the effect of IgG-Fc sialylation on this is conflicting, showing either increased, decreased or no binding to C1q due to sialylation (128, 129, 133, 153, 159, 160). The relative abundance of bisected GlcNAc residues seems to have no effect on either FcγR- or complement-mediated activities (128). Other modifications, e.g. carbamylation of IgG antibodies (161), and omitting clipping of C-terminal lysins (162) negatively impact IgG hexamerization as well. However, little is known about the importance of the biological implications of these changes. Interestingly, it has been shown that glycosylation patterns between allotypes within subclasses are quite similar in terms of fucosylation and sialylation, but substantial differences in bisection and galactosylation were observed between IgG3 allotypes (3).

In addition to N297 glycan site in the C_H_2 domain, one IgG2 and several IgG3 allotypes also contain an additional site at position 392 in the C_H_3 domain (163). Although occupancy of a glycan at 297 is virtually 100%, the frequency and impact on antibody-mediated functions of *N*-linked glycan at the 392 position are unknown.

### Fab *N*-linked glycosylation

*N*-linked glycosylation sites in the V_H_ and V_L_ regions have been observed in 10-25% of all serum IgG (117), which can contribute to both antibody stability (164), and modulate antigen binding (116, 165). In comparison to Fc glycans, the IgG variable domain glycans contain low levels of fucose and higher proportions of sialic acids, bisecting GlcNAc and galactose (166). It has been demonstrated that the presence of Fab glycans on human monoclonal antibodies can increase Fab binding affinity up to two-fold in an antigen-dependent manner (116), notably on anti-citrullinated protein antibodies in rheumatoid arthritis (167). In general, IgG4 antibodies have a 2-fold higher propensity to acquire Fab glycans compared to the other IgG subclasses, or similar to what is observed in IgE (116, 117, 168). This indicates a differential selection pressure of *N*-linked glycosylation site acquisition during affinity maturation of B cells, which depends on the frequency of immunization, antigen type, antibody isotype, subclass, and the location within the V region (117, 164, 169).

*N*-linked glycosylation motifs are generally not encoded by germline V, D or J segments, however potential glycosylation sites, requiring only a single base pair change during affinity maturation to emerge, are present in the CDR loops (116, 170). Recently, *N*-linked Fab glycans have been demonstrated to negatively affect FcRn-mediated binding in cells and therefore also FcRn-mediated placental transport of IgG in humans. The mechanism seems to involve a direct steric hindrance with the bulky Fab glycan clashing with the cellular membrane, further exaggerating the steric effect imposed by the Fab region on binding to membrane associated FcRn (171, 172). Variable domain glycans are postulated to convey a selective advantage through interaction with lectins and/or microbiota (170). Furthermore, B cell receptors expressing variable domain glycans also stay longer on the B cell surface, enhance B cell activation, and may contribute to the breach of tolerance of autoreactive B cells autoimmune disease (173). Elevated levels of Fab glycosylation have now been reported for several types of autoantibodies (168, 174), perhaps enriched due to the continuous antigen-exposure.

### O-linked glycosylation

O-linked glycosylation sites have only been found in the hinge region of IgG3, but not in the other IgG subclasses (175). O-glycans contain a *N*-acetylgalactosamine (GalNAc) and galactose residues, which may be sialylated (**Figure 2F**). Approximately 10% of IgG3 derived glycopeptides from human polyclonal serum samples contain O-linked glycans (175). Each IgG3 can contain up to three O-linked glycans at threonine (T) residues at triple repeat regions (TH2-7, TH3-7, TH4-7) on each side of the hinge region (163, 175). The IgG3 hinge region has a high degree of surface accessibility (176), which may explain the lower degree of O-linked glycosylation observed in IgG3 allotypes with a shorter hinge region (175). Although the function of O-glycosylation is still unknown, it might aid to protect the antibody from proteolytic cleavage or maintain the extended conformation and flexibility of IgG3 hinge regions.

## Antibody protein engineering to impact effector functions

Besides natural relevant amino acid residues, many engineered mutations have been described that can either enhance, reduce, or abolish antibody interactions with complement, FcγRs and FcRn (**Table 2**).

**Table 2:** Amino acid modifications influencing effector molecule binding and function.

						Fc effector molecules																	Function							
Modification or mutation (EU numbering)	Abbreviation	Subclass				FcγRI		FcγRIIa				FcγRIIb		FcγRIIIa				FcγRIIIb		FcRn	C1q		ADCC		ADCP		CDC		t1/2	Reference
		IgG1	IgG2	IgG3	IgG4			R131		H131				V158		F158														
E233P		x																												(15)
L234F					x																									(14)
G236A	GA	x																												(16, 177)
G237A		x																												(16)
P238D		x																												(178)
S239A		x																												(179)
I253A		x																												(7, 179, 180)
S254A		x																												(179)
D265A		x																												(179)
S267E		x																												(181, 182)
H268F		x																												(181)
D270A		x																												(179, 183)
R292A		x																												(179, 184)
N297(A/Q/G)	NA	x																												(179, 182, 185)
S298N		x																												(179)
K322A		x																												(183)
S324T		x																												(181)
K326W		x																												(186)
A327Q		x																												(179)
L328E		x																												(178)
L328F		x																												(182)
P329A		x																												(179, 183)
P331S				x	x																									(14, 187)
I332E		x																												(177, 184)
E333A		x																												(183, 186)
K338A		x																												(183)
E345R	Arg345	x																												(7)
E380A		x																												(179)
E430G		x																												(188)
H433A		x																												(7)
N434A		x																												(7, 189)
N435W		x																												(189)
K439E		x																												(7)
S440K		x																												(7)
C221D/D222C		x																												(190)
S228P/L235E					x																									(191)
F234A/L235A					x																									(192)
L234A/L235A	LALA	x			x																									(17)
L234A/L235E		x																												(193)
L234A/G237A		x																												(194)
G236A/G237A	GAGA	x																												(16)
G236N/H268D		x																												(195)
G236R/L328R	RR	x																												(196, 197)
G236A/I332E	AE	x																												(177, 181)
K236W/E333S	KWES	x																												(186)
S239D/I332E	DE or SDIE	x																												(177, 181, 182, 198)
P247I/A339Q		x																												199)
T250Q/M428L	QL				x																									(200)
M252Y/T256D	YD	x																												(201)
T256D/T307Q	DQ	x																												(201)
T256D/T307W	DW	x																												(201)
P257I/Q311I	PIQI	x																												(202)
S267E/L328F	SE/LF	x																												(178, 182, 196)
H268F/S324T	FT or HFST	x																												(181)
S298G/T299A	Ga	x																												(203)
K326A/E333A		x																												(186)
K326M/E333S		x																												(186)
K326W/E333S	WS	x																												(186)
A330S/P331S		x																												(14)
E380A/N434A		x																												(179)
M428L/N434S	MN or LS	x																												(204)
H433K/N434F	HN or KF	x																												(205)
E233P/L234V/L235A		x																												(15, 183)
L234A/L235A/K322A		x																												(193)
L234F/L235E/K322A		x																												(206)
L234F/L235Q/K322Q	FQQ	x																												(207, 208)
L234A/L235A/P329G	LALAPG	x																												(208)
L234F/L235E/P331S	FES	x																												(206, 208)
L234S/L235T/G236R		x																												(208)
L234A/L235A/G237A		x																												(208)
L234F/L235E/D265A	FEA	x																												(209)
L234Y/G236W/S298A	YWA	x																												(210)
L235A/G237A/E318A		x																												(211)
G236A/S239D/I332E	GASDIE	x																												(177, 184)
G236A/A330L/I332E	GAALIE	x																												(212)
S239D/S298A/I332E		x																												(198)
S239D/A330L/I332E	SDALIE or DLE	x																												(198)
T250Q/M428L/N434S	QLS	x																												(204, 213)
M252Y/S254T/T256E	YTE or MST	x																												(214, 215)
I253A/H310A/H435A	IHH	x																												(216)
P257I/M428L/N434S		x																												(204, 213)
V259I/N315D/N434Y	C6A-74	x																												(217, 218)
S267E/H268F/S324T	EFT	x																												(181)
H285D/T307Q/A378V	DQV	x																												(219)
S298A/E333A/K334A	AAA	x																												(179)
T307A/E380A/N434A		x																												(179, 220)
L309D/Q311H/N434S	DHS	x																												(221)
A327G/A330S/P331S		x																												(15)
I332E/M428L/N434S		x																												(198, 204, 213)
E333A/M428L/N434S	ALS	x																												(204, 213)
E345R/E430G/S440Y	RGY	x																												(7)
D376V/M428L/N434S		x																												(202, 204, 213)
E380A/M428L/N434S		x																												(179, 204, 213)
L234A/L235A/N297A/P329G		x																												(16)
L234A/L235A/M428L/N434S		x																												(204, 213)
G236A/S239D/A330L/I332E	GASDALIE	x																												(182, 222, 223)
S239D/H268F/S324T/I332E		x																												(181)
S239D/I332E/M428L/N434S	SDIE LS	x																												(177, 198, 204, 213)
P257I/Q311I/M428L/N434S	PIQI LS	x																												(196, 204, 213)
S267E/L328F/M428L/N434S	SE/LF LS	x																												(204, 213)
H268F/S324T/M428L/N434S	HFST LS	x																												(181; 204; 213)
H268Q/V309L/A330S/P331S	IgG2m4		x																											(224)
T307A/E380A/M428L/N434S		x																												(204; 213)
E233P/L234V/L235A/ΔG236/S267K					x																									(225)
L235V/F243L/R292P/Y300L/P396L	VPLIL	x																												(226, 227)
F243L/R292P/Y300L/V305I/P396L	Variant 18(LPLIL)	x																												(226, 228, 229)
G236A/S239D/I332E/M428L/N434S		x																												(177, 184, 204, 213)
G236A/S267E/H268F/S324T/I332E	EFT-EA	x																												(181, 211)
S239D/S298A/I332E/M428L/N434S		x																												(204, 213)
S239D/A330L/I332E/M428L/N434S		x																												(184, 198, 204, 213)
M252Y/S254T/T256E/M428L/N434S	YTE LS	x																												(204, 213, 214)
M252Y/S254T/T256E/H433K/N434F	YTE-KF or MST/HN	x																												(214, 230)
S267E/H268F/S324T/M428L/N434S	SEHFST	x																												(181, 204, 213)
N315D/A330V/N361D/A378V/N434Y	T5A-74	x																												(217, 218)
E345R/E430G/S440Y/M428L/N434S	RGY LS	x																												(7, 204, 213)
G236A/S239D/A330L/I332E/M428L/N434S		x																												(203, 204, 213)
M252Y/ S254T/T256Y + S239D/A330L/I332E	YTE-SDALIE	x																												(214)
E233D/G237D/P238D/H268D/P271G/A330R	V12	x																												(178)
E233P/L234V/L235A/ΔG236 + A327G/A330S/P331S		x																												(15, 183)
L234A/L235A/G237A/P238S/ H268A/A330S/P331S	IgG2c4d		x																											(231, 232)
V234A/G237A/P238S/H268A/V309L/A330S/P331S	IgG2σ		x																											(194)
G236A/S267E/H268F/S324T/I332E/M428L/N434S	EFT-EA LS	x																												(181, 204, 213)

black = blocking, red = decreased, grey = unchanged, blue = increased.

### Mutations to modulate complement binding

**Figure 4:**
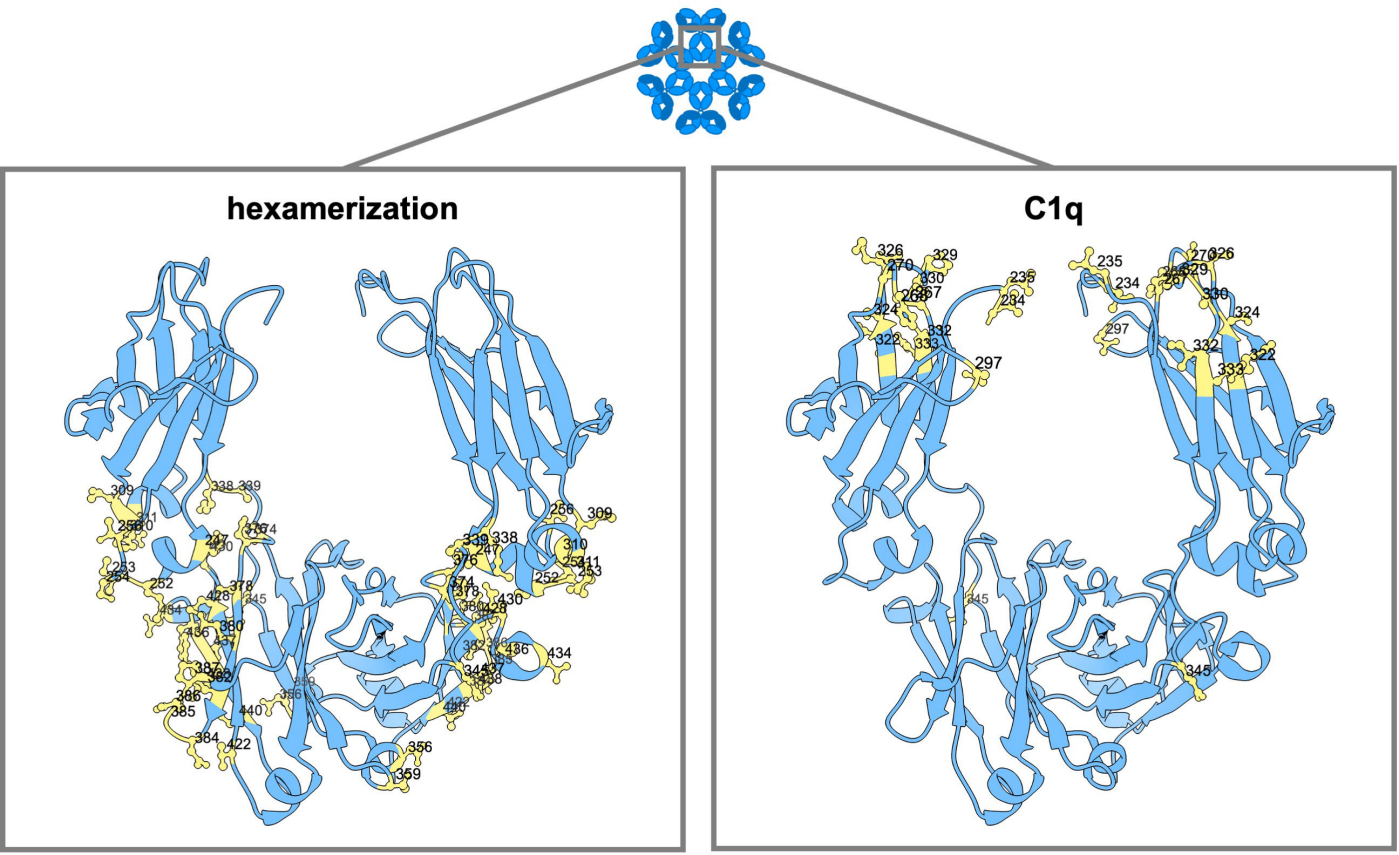
Amino acid mutation sites with impact on IgG hexamerization and C1q binding. Ribbon structure of dimeric IgG-Fc regions with highlighted amino acids involved in antibody hexamerization and Fc interaction with complement (C1q).

Two main ways of influencing complement activation are on the one hand affecting hexamerization, and on the other hand direct impact on C1q binding. Multiple mutations have been described that impact complement activation in either or both ways (**Figure 4**).

In the work of Strasser et al., the dynamics of IgG hexamer formation was studied by atomic force microscopy using an artificial surface (10, 11). They describe two different pathways to recruit IgG molecules into hexamers: from solution and through lateral diffusion of IgG-bound surface antigens in the cellular membrane (10, 11). At least four C1q globular heads, meaning engagement with four IgG antibodies, seem to be needed for efficient complement activation (9). Extensive binding subsites were found at the C_H_2 domains of IgG for the binding of the globular heads of C1q, one at the FG loop at position 325-331 (also important for the binding of FcγRs), one at the BC loop at position 266-272, and one at the DE loop at position 294-300 (9). The amino acid residues within these regions most critical for the binding of C1q are D270, K322, K326, P329, P331 (naturally a serine in IgG4 antibodies), E333 and K334 (183, 186, 233). Targeting these residues with point mutations, such as D270A (5), P329G/A (5, 183) or K322A (183, 233) can abolish C1q binding. Additionally, the G236/7A (GAGA) variant of IgG1 has been found to abrogate binding to C1q and downstream effector functions completely (16). K326W, K326W+E333S (WS), S267E+H268F+S324T (EFT) or S267E+H268F+S324T+G236A+I332E (EFT+AE) can increase C1q binding and CDC activity up to 47-fold (181, 193, 234). Another variant, comprising of the C_H_1 and hinge regions of IgG1 along with the Fc portion of IgG3 (1133), exhibited enhanced CDC that exceeded wild-type levels without gaining full recovery by protein A binding (101). Reconstituting the C-terminal part of the (IgG3-derived) C_H_3 domain with that of IgG1 again (113F), eliminated the protein A binding deficiency without compromising the enhanced CDC activity (101).

In addition to mutations that directly enhance C1q, amino acids outside this direct region have also been found that promote Fc : Fc interactions (188). Only very few fulfill the needs essential to use this for therapeutic antibody development (no multimer formation in solution and good pharmacokinetic properties). Variants E430G and E345K have been selected and are exploited as HexaBody Technology (188). Several studies have demonstrated that incorporating either one of these single mutations leads to increased complement activation and CDC of numerous cell types (133, 235–240). The single point mutations K439E and S440K can each be used to inhibit Fc : Fc interactions and subsequent complement activation via charge repulsion, which can in turn be overcome by using double mutants or mixtures containing each mutants (7, 133). The use of two variants which complement each other to gain function is the basis of the conceptual idea for the HexElect technology (241), in which hexamerization and subsequent complement activation is made dependent on the expression of two targets on the same cell. Although the hexamerization of IgG antibodies primarily occurs after antigen binding, the triple mutation E345R+E430G+S440Y (RGY) allows hexamerization in solution in a concentration and pH-dependent manner (7, 8, 188, 242).

### IgG-Fc substitutions to modulate FcγR binding

**Figure 5:**
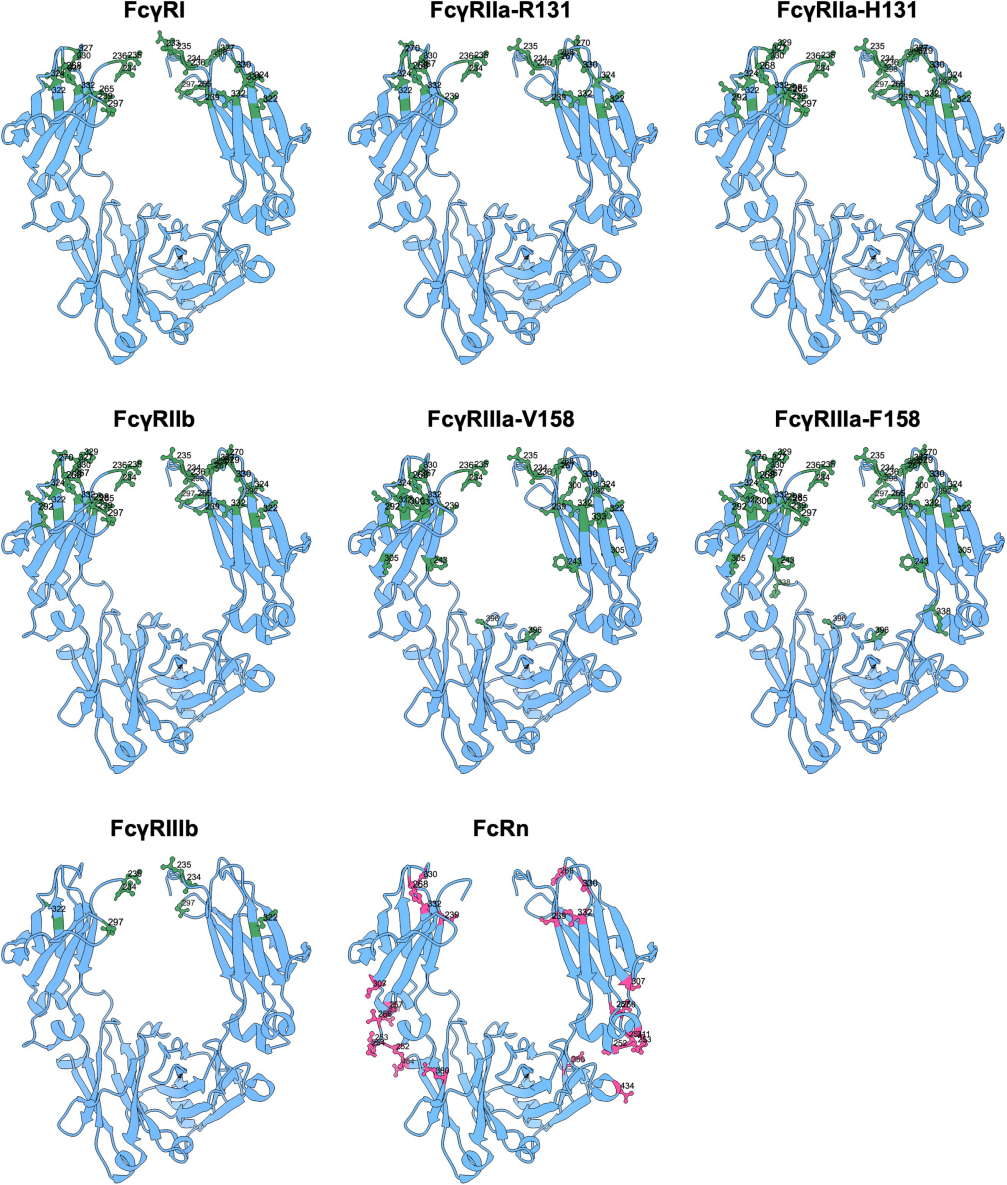
Amino acid mutation sites with impact on FcγR and FcRn binding. Ribbon structure of dimeric IgG-Fc regions with highlighted amino acids involved in FcγR and FcRn binding.

Many mutations have been described for the modulation of FcγR binding (**Table 2**; **Figure 5**).

IgG2 Abs, which naturally only bind FcγRIIa and lack G236, gain binding to both FcγRI and FcγRIIIa after insertion of glycine at position 236 (16). Likewise, IgG1 antibodies lacking G236 lose binding to all FcγRs (except for reduced binding to FcγRI) (16, 177). Variants of IgG1 expressing alanine instead of glycine at the adjacent position 237 have reduced FcγRI affinity and reduced FcγRIIIa-mediated ADCC (243), which also highlights the overall importance of the double G at position 236-7 in IgG for FcγR interaction. Another mutation of IgG1 in the lower hinge region that reduces binding to FcγRI due to antibodies which blocked the functions of active antibodies is E233P (15).

The G236A mutation in combination with S239D/I332E (DE) mutations in the IgG1 antibody, increases the binding affinity to FcγRI (3-fold), FcγRIIa (70-fold), FcγRIIb (13-fold) and FcγRIIIa (31-fold) (177). Despite the enhanced affinity to the inhibitory FcγRIIb, this triple mutation enhanced ADCP by macrophages (177). The addition of A330L to G236A/S239D/I332E (GASDALIE) resulted in increased affinity to FcγRIIa (25-fold) and FcγRIIIa F158 (30-fold), while FcγRIIb affinity was only slightly increased (182, 223).

The H268F/S324T (FT) double substitution resulted in decreased FcγRI, FcγRIIa-131R and FcγRIIb binding (181). However, the addition of the DE and G236A/I332E (AE) substitutions to the FT variant improved FcγR binding considerably. The combination of FT + DE resulted in enhanced binding to FcγRs, particularly to FcγRIIIa, whereas the combination of FT + AT resulted in selective enhancement for FcγRIIa and FcγRIIIa binding. These variants enhanced effector functions accordingly (ADCC: up to 22-fold; ADCP: up to 4.7-fold). The S267E/H268F + S324T variant (EFT) increased binding to FcγRIIa-R131 and FcγRIIb significantly. Combination with the AE substitution produced a variant (EFT + AE) with increased FcγRIIa affinity and FcγRIIIa binding slightly better than native IgG1 (181). Whereas, the amino acid substitutions S267E/L328F when introduced into IgG1, resulted in increased affinity to FcγRIIb (430-fold) with minimal changes in binding to FcγRI, FcγRIIa-131H and no binding to FcγRIIa-158V (196). The mutation P238D also enhanced binding to FcγRIIb, while either completely abolishing or significantly reducing binding to FcγRI, FcγRIIa-131H and FcγRIIa-158V (178). This mutation (P238D) in combination with five additional amino acid substitutions E233D/G237D/H268D/P271G/A330R (termed V12) enhanced binding to FcγRIIb even more (~217-fold) (178, 211, 244). Other sets of substitutions described to enhance IgG1 binding to FcγRIIb encompass G236N/H268D and G236N/H268D/A330K, which are abbreviated V2 and V3, respectively (195). IgG1 with V12-, V2- or V3- bearing Fcs were found to engage FcγRIIb for its rather recently discovered recycling function (244), and demonstrated efficient soluble target clearance *in vivo* when combined with antigen-sweeping Fabs (195, 244, 245). Strongly reduced binding to FcγRIIa-131R, but also to both FcγRIIb and FcγRIIa-158F, can be achieved by a single mutation D270A. This mutation does not affect binding to either FcγRI, FcγRIIa-131H, or FcRn (179).

Both the combination of S298A, E333A, and K334A mutations (AAA) in an IgG1 antibody in combination with DE improved the affinity to both allotypes of FcγRIIIa (179, 198). However, an increase in binding to the inhibitory FcγRIIb was also observed with the DE double mutant (198). By the addition of a leucine at position 330, S239D/A330L/I332E (DLE), this effect was reversed. The DLE mutations cause an open conformation of the Fc by separating the two C_H_2 by introducing additional hydrogen bonds between S239D/I332E in the Fc and lysine at position 158 in the FcγRIIIa (206). In addition, Mimoto et al. showed that antibodies with mutations L234Y, G236W, and S298A (YWA), in one heavy chain and DLE in the other, mediated ADCC of tumor antigen-expressing cells at a higher capacity than antibodies that contained only the YWA or DLE mutations (210). Combinations of F243L, R292P, Y300L, V305I, and P396L (variant 18) mutations had an improved Fc binding to FcγRIIa and FcγRIIIa (10-fold) without increasing binding to the inhibitory FcγRIIb receptor (<2-fold), resulting in >100-fold increase ADCC activity (226, 228, 229). However, this combination of mutations has evolved to L235V, F243L, R292P, Y300L, and P396L in follow-up studies (227, 246).

To reduce or prevent IgG binding to FcγRs, a single mutation of leucine to glutamic acid at position 235 is sufficient for reduced binding (100-fold) to the FcγRs on U937 cells (247). The removal of G236 in IgG1 abrogates binding to all FcγR, with negligible trace binding still detectable for FcγRI and minor effect on C1q binding and complement activity (16). A refined IgG1 variation was found which comprises the combination of L234A and L235A (LALA), which reduced detectable binding to FcγRI, FcγRIIa, and FcγRIIIa, but also complement, significantly (192, 248). The use of LALA appears to be more effective than either L234A or L235A alone and only in combination, low to undetectable binding to the high affinity Fc receptor FcγRI can be achieved (14). Although the LALA mutations do not completely abrogate FcγR binding, several antibodies have by now been approved with this Fc configuration, for example the humanized anti-IL36R and anti-CD3 IgG1-based antibodies spesolimab and teplizumab, respectively (249). The LALA mutations have also provided a foundation for the modification of other mutations. LALA in combination with P331S can eliminate binding to FcγRs completely without disrupting the overall conformation of the Fc (206, 208). Similarly, LALA combined with glycine at position 329 (LALA-PG) was an enhancement over LALA mutations alone as the combination eliminated all Fc-mediated effector functions, including complement (191, 250). The LALA-PG containing bispecific anti-CD20 x CD3 IgG1 glofitamab was approved for the treatment of relapsed or refractory diffuse large B cell lymphoma (251). Moreover, more than ten monoclonal antibodies with the same Fc configuration are currently in clinical development (252). Similarly, the LALA-PG mutations with a N297A substitution, resulting in non-glycosylated Fc, also have no detectable binding to any FcγR (16, 208). On top of that, Engelberts et al. showed that the combination of L234F/L235E/D265A resulted in no detectable binding to FcγRI, and reduced binding to both the low affinity FcγRs and C1q (209, 253). In addition, the mutations G236R/L328R either reduced or completely abrogated binding to the FcγRs (FcγRIIa-131R and FcγRIIa-158F were not assessed) and the S267E substitution reduced binding for all low affinity FcγRs (196). A substitution to lysine at position 267 combined with a series of E233P/L234V/L235A mutations and a deletion of residue G236 showed a lack of binding to all FcγRs (FcγRIIa-158F was not assessed) (225). Together, this points to a crucial role of this lower hinge region to regulate the binding to FcγRs.

Other crucial mutations in various combinations that can eliminate FcγRI, FcγRIIa, FcγRIIb, and FcγRIIIa binding include proline 233, alanine 237, and alanine 318 (179, 250). Glycine 237 and glutamic acid 318 are both essential for FcγRII binding (14). Another double mutation is S228P and L235E (SPLE or PE) (254). The SPLE mutations have been introduced into IgG4, although IgG4 has low binding to FcγRs in general due to the phenylalanine at position 234 (255). In addition, it has been shown that S228P stabilizes the structure of IgG4 and prevention of FAE *in vivo*. The Fc variant of IgG2 (referred to as IgG2σ), with V234A/G237A/P238S/H268A/V309L/A330S/P331S substitutions showed no binding to FcγRs and C1q, resulting in the total lack of inducing any immune effector functions (194).

As mentioned before, an alternative strategy to minimize effector functions is to remove the *N*-linked glycosylation site at amino acid residue 297 in the C_H_2 domain (256). Point mutations, such as N297A, N297Q and N297G (257), have all been used to this end and N297A and N297G have been incorporated in approved therapeutic mAbs, such as atezolizumab (anti-PD-L1) and mosunetuzumab (anti-CD3xCD20), respectively (258, 259). Interestingly, there have been multiple studies attempting to re-engineer effector function into aglycosylated Fc domains, in order to facilitate the use of prokaryotic expression systems. They showed that engineered deglycosylated Fc variants including substitutions at S298G/T299A (203), N297D/S298T/K326I/A327Y/L328G (DTT-IYG) (260), S298G/T299A/K326I/A327Y/L328G/E382V/N390D/M428L (AglycoT-Fc1004-IYG) (261), and E382V/M428I (262, 263) restore FcγR binding and effector functions.

### IgG-Fc substitutions to modulate FcRn binding

Reduced binding to human FcRn was observed, when each of the interface residues I253, H310 and H435 was substituted to alanine (264). Combining the three substitutions I253A, H310A and H435A (IHH) in an IgG1 completely abrogates binding to human FcRn (216). The IHH and the H435A variants are now commonly used as non-FcRn binding variants (216, 265, 266)

Efforts have also been made to influence the IgG-FcRn interaction to increase serum half-life. A study used rationally designed libraries targeting the IgG-FcRn interface around residues 252 to 256 and 433 to 436, which among others identified an IgG1 Fc variant, bearing M252Y, S254T and T256E (YTE) substitutions. This YTE variant exhibited stronger binding to human FcRn (4-fold) at acidic pH without increased binding at neutral pH, and therefore causing increased antibody half-life and serum persistence (214, 215, 267). The YTE-mutated anti-respiratory syncytial virus (RSV) IgG1 nirsevimab has recently been approved as prevention therapy for young children at risk, exhibiting a reported serum half-life of 71 days (268). Following this rational, different variants have been discovered to enhance *in vivo* half-life of IgG antibodies, all based on the same conceptual principle: increasing the binding to FcRn at acidic pH and efficient release at neutral pH. An IgG1 variant with H433K and N434F (KF) substitutions (205) showed enhanced binding to FcRn at endosomal pH (16-fold) and extended half-life in nonhuman primates (NHP) (269). A similar pH-dependent affinity to both human and NHP FcRn was found for the single substitution at position 434 from either asparagine to alanine (~2-fold) or to tryptophan (80-fold), which exhibited prolonged half-life in NHPs compared to wild type IgG (189). Another IgG Fc variant proven to both exhibit 11-fold enhancement in binding affinity to FcRn and extend half-life in humans bears M428L and N434S substitutions (LS) (270).

Again, the extended half-life of the LS variant is attributed to the reduced off-rate at pH 6.0 and without increasing binding to FcRn at neutral pH. However, an increased *in vivo* efficacy might depend on the disease model system and target kind, given that the same variant in human FcRn and FcγR transgenic mice did not outperform wild type IgG, despite exhibiting significantly longer half-life (271). Therapeutic antibodies containing an LS variant substituted Fc is are being evaluated in multiple human clinical trials (270). The anti-C5 complement inhibitor ravulizumab was recently approved (272), exhibiting a serum half-life of 56.6 days in patients with generalized myasthenia gravis (273). In addition, The LS variant has also been incorporated in neutralizing monoclonal antibodies (tixagevimab, cilgavimab amubarvimab and romtusevimab) against severe acute respiratory syndrome coronavirus 2 (SARS-CoV-2) (274, 275), all with an extended half-life.

Comparable FcRn binding profiles were described for the single amino acid substitution M428L and the combination of T250Q/M428L, which both achieved significantly slower clearance compared to wild type IgG in rhesus monkeys (276). T250Q/M428L and V308P bearing IgG4s have been found to exhibit prolonged *in vivo* half-life in cynomolgus monkeys (277). Another effort resulted in the identification of three double mutant variants with a half-life comparable to that of the LS variant in both human FcRn transgenic mice and cynomolgus monkeys, comprising either M252Y/T256D (YD), T256D/T307Q (DQ) or T256D/T307W (DW) substitutions (201). Shortly after, the triple mutant L309D, Q311H and N434S (DHS) was described, which was validated in a human FcRn, FcγR and IgG1 transgenic mouse model and even outperformed the YTE and LS variants regarding their half-life (221). However, the prolonged half-life of the YTE (267) and KF (205, 269) variants in combination (YTE-KF or MST-HN) exhibits increased binding to FcRn (20-fold) at both low (pH ≤ 6.5) and neutral pH (278). This YTE-KF variant binds FcRn strong enough to antagonize the IgG-FcRn interaction and was found to specifically reduce IgG levels *in vivo*. Such an Fc fragment is called an Abdeg (antibody that enhances IgG degradation) (279), which is being clinically investigated under the name efgartigimod for the treatment of IgG-mediated autoimmune diseases and has recently been approved for the treatment of generalized myasthenia gravis (280–282). Other variants with enhanced binding to FcRn at both physiological and acidic pH include M252Y/N286E/N434Y (YEY) and M252Y/V308P/N434Y (YPY) (283), which have been found to utilize FcRn for improved soluble target clearance in NHP when combined with antigen sweeping Fabs (284).

Whereas FcγR binding and *N*-linked glycosylation are generally thought to not affect *in vivo* half-life of IgG (285) and not to affect transport across the placenta (286), a single amino acid deletion at position 294 in IgG1 (ΔE294) was found to result in hypersialylated variants, which exhibited longer half-life than their non-ΔE294 counterparts in human FcRn transgenic mice (218). This was also confirmed for variants bearing half-life extension substitutions found by the same group, such as V259I/N315D/N434Y (C6A-74) and N315D/A330V/N361D/A378V/N434Y (T5A-74) (217, 218). Other than IgG Fc mutations which affect the interaction of IgG with FcRn or FcRn-mediated processes, several groups have found an impact of the Fabs (171), Fab glycans (172), possibly their flexibility is also involved (50), physicochemical properties (109, 287, 288), and antigen-binding on FcRn binding and cellular transport (289, 290).

It is important to highlight that amino acid substitutions, such as LS or YTE, which affect FcRn binding, often also influence the interaction with FcγR (and C1q) and thereby their effector function profile, which is a critical consideration to make depending on therapeutic target and purpose (219, 221, 291).

## Outlook: therapeutical antibodies

The number of novel antibody-based molecules undergoing a first regulatory review is at a record level but the number of approved drugs has not kept pace (292). Although we have learned a lot about specific amino acid residues that are required for enhancing or inhibiting antibody-mediated effector functions, the development of antibody-based therapeutics to counteract biological processes may require more than just modifying the sequence of antibody-based molecules to modulate its interaction with the host immune system (293). It has been shown that IgG variants without core fucosylation cause elevated ADCC, through increased FcγRIIIa affinity (56, 127, 128, 146), which has resulted in next-generation glyco-engineered therapeutic antibodies that lack core fucosylation for targeting tumors (292). Some examples include mogamulizumab (KW-0761, AMG-761), which is an afucosylated humanized IgG1 targeting CC chemokine receptor 4 for the treatment of patients with relapsed or refractory adult T-cell lymphoma (294). Another approved afucosulated humanized IgG1 is benralizumab (MEDI-563) which engages IL-5 Receptor α-chain in the treatment of severe eosinophilic asthma (295).

Currently only IgG1, IgG2 and IgG4 antibodies are being considered for therapeutic purposes, but especially IgG3 (most potent subclass in mediating Fc effector functions) and IgG polymorphisms could be utilized to enhance antibody-mediated effector function via to differential binding to endogenous FcγRs and complement proteins (93). Studies have found that monoclonal antibodies with identical variable regions, but different IgG subclasses and allotypes can mediate enhanced Fc effector function, modulated by the hinge length and naturally occurring amino acid substitutions (3, 81, 98). IgG3 is currently not used for any therapeutic antibodies due to its short half-life and large number of alleles which may result in anti-allotypic effects. However, IgG3 variants such as IGHG3*17, *18, and *19 have a half-life equivalent to IgG1 and there is no evidence that allelic mismatch causes any clinical adverse effects (81, 91, 296, 297). Therefore, next generation therapeutic antibodies should consider utilizing IgG3 backbones due to the greater molecular flexibility and stronger affinity to FcγRs and C1q.

Despite being the least abundant IgG in human plasma, IgG4 antibodies, but also mutations such as LALA, are used therapeutically when weak or ‘silenced’ effector functions are needed. As of 2019, more than 30 biopharmaceuticals with an IgG4-based Fc fragment were approved or were in late-stage clinical development (298). Recently, bispecific monoclonal antibodies have emerged as a growing new class of therapeutics with a wide spread of bispecific antibodies across all stages of clinical trials and platforms (259, 299), some of which were inspired by IgG4 FAE (300–302). Other current areas of development of improved IgG4-based therapeutics focuses on half-life modulation, stability, and inhibiting downstream processes. For example, it has been demonstrated that IgG4 monoclonal antibodies with the S228P/L235E/R409K mutations, not capable of half-molecule exchange, showed less of a tendency to aggregate at low pH than S228P/L235E variants and stabilization of IgG4 antibodies in non-exchanging formats (303). The E357Q/S364K-L368D/K370S variant resulted in a C_H_3 region that remained more stable than that of native IgG4 (225). The YTE mutations seem to have the most improved pharmacokinetic properties in combination with reduced effector functions (267, 304).

Although it is possible to learn through natural and engineered Fc modifications, new approaches are needed to consider and screen variants containing multiple mutations, asymmetric binding modes, glycan profiles and effector molecule binding, especially when hexamerization or bispecific antibodies come in play (305, 306). The antibody modifications should be substituted into next generation therapeutic antibodies, but also in monitoring of therapeutically administered IgG (307). Understanding the key determinants that shape antibody-mediated functions is crucial in designing more effective antibody-based therapeutics. Therefore, exploring the associations between IgG allotypes and/or glycan profiles and effector functions, including poorly understood mechanisms such as TRIM21 binding, could open new strategies in the development of therapeutic antibodies. With growing understanding of antibody-mediated immune responses and the constant development of molecular technologies, the future for tailored and effective antibody-based therapeutics seems limitless.

## Author contributions

TD: Writing – original draft, Writing – review & editing. MB: Writing – original draft, Writing – review & editing. TvO: Writing – original draft, Writing – review & editing. JS: Writing – review & editing. AL: Writing – review & editing. TR: Writing – review & editing. GV: Writing – review & editing.

## References

[1] Rogentine GN Rowe DS Bradley J.A. Waldmann TA Fahey JL. Metabolism of human immunoglobulin D (IgD). J Clin Invest (1966) 45:1467–78. doi: 10.1172/JCI105454 PMC292826 5919348

[2] Waldmann TA Iio A Ogawa M McIntyre OR Strober W. The metabolism of IgE: studies in normal individuals and in a patient with IgE myeloma. J Immunol (1976) 117:1139–44. doi: 10.4049/jimmunol.117.4.1139 977946

[3] de Taeye S Bentlage AEH Mebius MM Meesters JI Lissenberg-Thunnissen S Falck D. FcγR binding and ADCC activity of human IgG allotypes. Front Immunol (2020) 11:740–0. doi: 10.3389/fimmu.2020.00740 PMC7218058 32435243

[4] de Taeye SW Rispens T Vidarsson G. The ligands for human IgG and their effector functions. Antibodies (2019) 8:30–0. doi: 10.3390/antib8020030 PMC6640714 31544836

[5] Michaelsen TE Sandlie I Bratlie DB Sandin RH Ihle O. Structural difference in the complement activation site of human IgG1 and IgG3. Scandinavian J Immunol (2009) 70:553–64. doi: 10.1111/j.1365-3083.2009.02338.x 19906198

[6] Oskam N Damelang T Streutker M Ooijevaar-de Heer P Nouta J Koeleman C. Factors affecting IgG4-mediated complement activation. Front Immunol (2023) 14. doi: 10.3389/fimmu.2023.1087532 PMC9910309 36776883

[7] Diebolder CA Beurskens FJ De Jong RN Koning RI Strumane K Lindorfer MA. Complement is activated by IgG hexamers assembled at the cell surface. Science (2014) 343:1260–3. doi: 10.1126/science.1248943 PMC4250092 24626930

[8] Wang G de Jong RN van den Bremer ETJ Beurskens FJ Labrijn AF Ugurlar D. Molecular basis of assembly and activation of complement component C1 in complex with immunoglobulin G1 and antigen. Mol Cell (2016) 63:135–45. doi: 10.1016/j.molcel.2016.05.016 27320199

[9] Ugurlar D Howes SC de Kreuk B-J Koning RI de Jong RN Beurskens FJ. Structures of C1-IgG1 provide insights into how danger pattern recognition activates complement. Science (2018) 359:794–7. doi: 10.1126/science.aao4988 29449492

[10] J.r. Strasser RN Beurskens FJ Wang G Heck AJR Schuurman J Parren PWHI. Unraveling the macromolecular pathways of IgG oligomerization and complement activation on antigenic surfaces. Nano Lett (2019) 19:4787–96. doi: 10.1021/acs.nanolett.9b02220 31184907

[11] J.r. Strasser de Jong RN Beurskens FJ Schuurman J Parren PWHI Hinterdorfer P. Weak fragment crystallizable (Fc) domain interactions drive the dynamic assembly of IgG oligomers upon antigen recognition. ACS nano (2019) 14:2739–50. doi: 10.1021/acsnano.9b08347 31887016

[12] Goldberg BS Ackerman ME. Antibody-mediated complement activation in pathology and protection. Immunol Cell Biol (2020) 98:305–17. doi: 10.1111/imcb.12324 PMC7293394 32142167

[13] Abendstein L Dijkstra DJ Tjokrodirijo RTN van Veelen PA Trouw LA Hensbergen PJ. Complement is activated by elevated IgG3 hexameric platforms and deposits C4b onto distinct antibody domains. Nat Commun (2023) 14:4027. doi: 10.1038/s41467-023-39788-5 37419978 PMC10328927

[14] Lund J Winter G Jones PT Pound JD Tanaka T Walker MR. and Fc gamma RII interact with distinct but overlapping sites on human IgG. J Immunol (Baltimore Md.: 1950) (1991) 147:2657–62. 1833457

[15] Armour KL Clark MR Hadley AG Williamson LM. Recombinant human IgG molecules lacking Fcγ receptor I binding and monocyte triggering activities. Eur J Immunol (1999) 29:2613–24. doi: 10.1002/(SICI)1521-4141(199908)29:08<2613::AID-IMMU2613>3.0.CO;2-J 10458776

[16] Brinkhaus M Douwes RGJ Bentlage AEH Temming AR de Taeye SW Tammes Buirs M. Glycine 236 in the lower hinge region of human igG1 differentiates fcγR from complement effector function. J Immunol (Baltimore Md. 1950) (2020) 205:3456–67. doi: 10.4049/jimmunol.2000961 33188070

[17] Bruhns P Iannascoli B England P Mancardi DA Fernandez N Jorieux S. Specificity and affinity of human Fcγ receptors and their polymorphic variants for human IgG subclasses. Blood J Am Soc Hematol (2009) 113:3716–25. doi: 10.1182/blood-2008-09-179754 19018092

[18] Chung AW Navis M Isitman G Leia W Silvers J Janaki A. Activation of NK cells by ADCC antibodies and HIV disease progression. J acquired Immune deficiency syndromes (2011) 58(2):127–31. doi: 10.1097/QAI.0b013e31822c62b9 PMC3175260 21792067

[19] Ackerman ME Mikhailova A Brown EP Dowell KG Walker BD Bailey-Kellogg C. Polyfunctional HIV-specific antibody responses are associated with spontaneous HIV control. PloS Pathog (2016) 12:e1005315–e1005315. doi: 10.1371/journal.ppat.1005315 26745376 PMC4706315

[20] Lu LL Chung AW Rosebrock TR Ghebremichael M Yu WH Grace PS. A functional role for antibodies in tuberculosis. Cell (2016) 167:433–43. doi: 10.1016/j.cell.2016.08.072 PMC5526202 27667685

[21] McLean MR Lu LL Kent SJ Chung AW. An inflammatory story: Antibodies in tuberculosis comorbidities. Front Immunol (2019) 10:2846–6. doi: 10.3389/fimmu.2019.02846 PMC6913197 31921122

[22] Wang TT Sewatanon J Memoli MJ Wrammert J Bournazos S Bhaumik SK. IgG antibodies to dengue enhanced for FcγRIIIA binding determine disease severity. Science (2017) 355:395–8. doi: 10.1126/science.aai8128 PMC5557095 28126818

[23] Damelang T Aitken EH Hasang W Lopez E Killian M Unger HW. Antibody mediated activation of natural killer cells in malaria exposed pregnant women. Sci Rep (2021) 11:4130–0. doi: 10.1038/s41598-021-83093-4 PMC7893158 33602987

[24] Yoshida M Claypool SM Wagner JS Mizoguchi E Mizoguchi A Roopenian DC. Human neonatal Fc receptor mediates transport of IgG into luminal secretions for delivery of antigens to mucosal dendritic cells. Immunity (2004) 20:769–83. doi: 10.1016/j.immuni.2004.05.007 15189741

[25] Ohsaki A Venturelli N Buccigrosso TM Osganian SK Lee J Blumberg RS. Maternal IgG immune complexes induce food allergen–specific tolerance in offspring. J Exp Med (2018) 215:91–113. doi: 10.1084/jem.20171163 29158374 PMC5748859

[26] Vidarsson G Stemerding AM Stapleton NM Spliethoff SE Janssen H Rebers FE. FcRn: an IgG receptor on phagocytes with a novel role in phagocytosis. Blood (2006) 108:3573–9. doi: 10.1182/blood-2006-05-024539 16849638

[27] Pyzik M Sand KMK Hubbard JJ Andersen JT Sandlie I Blumberg RS. The neonatal Fc receptor (FcRn): a misnomer? Front Immunol (2019) 10:1540–0. doi: 10.3389/fimmu.2019.01540 PMC6636548 31354709

[28] Cines DB Zaitsev S Rauova L Rux AH Stepanova V Krishnaswamy S. FcRn augments induction of tissue factor activity by IgG-containing immune complexes. Blood (2020) 135:2085–93. doi: 10.1182/blood.2019001133 PMC7273830 32187355

[29] Hubbard JJ Pyzik M Rath T Kozicky LK Sand KMK Gandhi AK. FcRn is a CD32a coreceptor that determines susceptibility to IgG immune complex–driven autoimmunity. J Exp Med (2020) 217:e20200359. doi: 10.1084/jem.20200359 32658257 PMC7537387

[30] Wilson TJ Fuchs A Colonna M. Cutting edge: human FcRL4 and FcRL5 are receptors for IgA and IgG. J Immunol (2012) 188:4741–5. doi: 10.4049/jimmunol.1102651 PMC3634363 22491254

[31] Li FJ Won WJ Becker EJ Easlick JL Tabengwa EM Li R. Emerging roles for the FCRL family members in lymphocyte biology and disease. Fc Receptors (2014) 382:29–50. doi: 10.1007/978-3-319-07911-0_2 PMC4242170 25116094

[32] Foss S Watkinson RE Grevys A McAdam MB Bern M Høydahl LS. TRIM21 immune signaling is more sensitive to antibody affinity than its neutralization activity. J Immunol (2016) 196:3452–9. doi: 10.4049/jimmunol.1502601 PMC4977002 26962230

[33] Foss S Bottermann M Jonsson A Sandlie I James LC Andersen JT. TRIM21—from intracellular immunity to therapy. Front Immunol (2019) 10:2049–9. doi: 10.3389/fimmu.2019.02049 PMC6722209 31555278

[34] Anthony RM Wermeling F Karlsson MCI Ravetch JV. Identification of a receptor required for the anti-inflammatory activity of IVIG. Proc Natl Acad Sci (2008) 105:19571–8. doi: 10.1073/pnas.0810163105 PMC2604916 19036920

[35] Sondermann P Pincetic A Maamary J Lammens K Ravetch JV. General mechanism for modulating immunoglobulin effector function. Proc Natl Acad Sci (2013) 110:9868–72. doi: 10.1073/pnas.1307864110 PMC3683708 23697368

[36] Nimmerjahn F Vidarsson G Cragg MS. Effect of posttranslational modifications and subclass on IgG activity: from immunity to immunotherapy. Nat Immunol (2023) 24:1244–55. doi: 10.1038/s41590-023-01544-8 37414906

[37] Yu X Vasiljevic S Mitchell DA Crispin M Scanlan CN. Dissecting the molecular mechanism of IVIg therapy: the interaction between serum IgG and DC-SIGN is independent of antibody glycoform or Fc domain. J Mol Biol (2013) 425:1253–8. doi: 10.1016/j.jmb.2013.02.006 23416198

[38] Temming AR Dekkers G van de Bovenkamp FS Plomp HR Bentlage AEH Szittner Z. Human DC-SIGN and CD23 do not interact with human IgG. Sci Rep (2019) 9:1–10. doi: 10.1038/s41598-019-46484-2 31292524 PMC6620288

[39] Rostamzadeh D Kazemi T Amirghofran Z Shabani M. Update on Fc receptor-like (FCRL) family: new immunoregulatory players in health and diseases. Expert Opin Ther Targets (2018) 22:487–502. doi: 10.1080/14728222.2018.1472768 29737217

[40] Franco A Damdinsuren B Ise T Dement-Brown J Li H Nagata S. Human Fc receptor–like 5 binds intact IgG *via* mechanisms distinct from those of Fc receptors. J Immunol (2013) 190:5739–46. doi: 10.4049/jimmunol.1202860 PMC3660407 23616577

[41] James LC Keeble AH Khan Z Rhodes DA Trowsdale J. Structural basis for PRYSPRY-mediated tripartite motif (TRIM) protein function. Proc Natl Acad Sci (2007) 104:6200–5. doi: 10.1073/pnas.0609174104 PMC1851072 17400754

[42] Keeble AH Khan Z Forster A James LC. TRIM21 is an IgG receptor that is structurally, thermodynamically, and kinetically conserved. Proc Natl Acad Sci (2008) 105:6045–50. doi: 10.1073/pnas.0800159105 PMC2329685 18420815

[43] Mukadam AS Miller LVC Smith AE Vaysburd M Sakya SA Sanford S. Cytosolic antibody receptor TRIM21 is required for effective tau immunotherapy in mouse models. Science (2023) 379:1336–41. doi: 10.1126/science.abn1366 PMC7614512 36996217

[44] Jackson KJL Wang Y Collins AM. Human immunoglobulin classes and subclasses show variability in VDJ gene mutation levels. Immunol Cell Biol (2014) 92:729–33. doi: 10.1038/icb.2014.44 24913324

[45] Potter M. Structural correlates of immunoglobulin diversity. Survey immunologic Res (1983) 2:27–42. doi: 10.1007/BF02918394 6417753

[46] Hsieh F-L Higgins MK. The structure of a LAIR1-containing human antibody reveals a novel mechanism of antigen recognition. Elife (2017) 6:e27311–1. doi: 10.7554/eLife.27311 PMC5459573 28527239

[47] Roux KH Strelets L Michaelsen TE. Flexibility of human IgG subclasses. J Immunol (Baltimore Md.: 1950) (1997) 159:3372–82. 9317136

[48] Jay JW Bray B Qi Y Igbinigie E Wu H Li J. IgG antibody 3D structures and dynamics. Antibodies (2018) 7:18–8. doi: 10.3390/antib7020018 PMC6698877 31544870

[49] Dillon TM Ricci MS Vezina C Flynn GC Liu YD Rehder DS. Structural and functional characterization of disulfide isoforms of the human IgG2 subclass. J Biol Chem (2008) 283:16206–15. doi: 10.1074/jbc.M709988200 PMC3259628 18339626

[50] Stapleton NM Brinkhaus M Armour KL Bentlage AEH de Taeye SW Temming AR. Reduced FcRn-mediated transcytosis of IgG2 due to a missing Glycine in its lower hinge. Sci Rep (2019) 9:7363–3. doi: 10.1038/s41598-019-40731-2 PMC6517591 31089170

[51] Michaelsen TE Næss LM Aase A. Human IgG3 is decreased and IgG1, IgG2 and IgG4 are unchanged in molecular size by mild reduction and reoxidation without any major change in effector functions. Mol Immunol (1993) 30:35–45. doi: 10.1016/0161-5890(93)90424-A 8417373

[52] Bashirova AA Zheng W Akdag M Augusto DG Vince N Dong KL. Population-specific diversity of the immunoglobulin constant heavy G chain (IGHG) genes. Genes Immun (2021) 22:327–34. doi: 10.1038/s41435-021-00156-2 PMC8674132 34864821

[53] Redpath S Michaelsen TE Sandlie I Clark MR. The influence of the hinge region length in binding of human IgG to human Fcγ receptors. Hum Immunol (1998) 59:720–7. doi: 10.1016/S0198-8859(98)00075-5 9796740

[54] Hagihara Y Saerens D. Engineering disulfide bonds within an antibody. Biochim Biophys Acta (BBA)-Proteins Proteomics (2014) 1844:2016–23. doi: 10.1016/j.bbapap.2014.07.005 25038323

[55] Wypych J Li M Guo A Zhang Z Martinez T Allen MJ. Human IgG2 antibodies display disulfide-mediated structural isoforms. J Biol Chem (2008) 283:16194–205. doi: 10.1074/jbc.M709987200 PMC3259661 18339624

[56] Vidarsson G Dekkers G Rispens T. IgG subclasses and allotypes: from structure to effector functions. Front Immunol (2014) 5:520–0. doi: 10.3389/fimmu.2014.00520 PMC4202688 25368619

[57] Lightle S Aykent S Lacher N Mitaksov V Wells K Zobel J. Mutations within a human IgG2 antibody form distinct and homogeneous disulfide isomers but do not affect Fc gamma receptor or C1q binding. Protein Sci (2010) 19:753–62. doi: 10.1002/pro.352 PMC2867015 20120022

[58] Yu X Orr CM Chan HTC James S Penfold CA Kim J. Reducing affinity as a strategy to boost immunomodulatory antibody agonism. Nature (2023) 614:539–47. doi: 10.1038/s41586-022-05673-2 36725933

[59] van Der Neut Kolfschoten M Schuurman J Losen M Bleeker WK Martínez-Martínez P Vermeulen E. Anti-inflammatory activity of human IgG4 antibodies by dynamic Fab arm exchange. Science (2007) 317:1554–7. doi: 10.1126/science.1144603 17872445

[60] Rispens T Davies AM Ooijevaar-de Heer P Absalah S Bende O Sutton BJ. Dynamics of inter-heavy chain interactions in human immunoglobulin G (IgG) subclasses studied by kinetic Fab arm exchange. J Biol Chem (2014) 289:6098–109. doi: 10.1074/jbc.M113.541813 PMC3937676 24425871

[61] Labrijn AF Rispens T Meesters J Rose RJ den Bleker TH Loverix S. Species-specific determinants in the IgG CH3 domain enable Fab-arm exchange by affecting the noncovalent CH3–CH3 interaction strength. J Immunol (2011) 187:3238–46. doi: 10.4049/jimmunol.1003336 21841137

[62] Davies AM Rispens T Ooijevaar-de Heer P Gould HJ Jefferis R Aalberse RC. Structural determinants of unique properties of human IgG4-Fc. J Mol Biol (2014) 426:630–44. doi: 10.1016/j.jmb.2013.10.039 PMC3905167 24211234

[63] Labrijn AF Buijsse AO Van den Bremer ETJ Verwilligen AYW Bleeker WK Thorpe SJ. Therapeutic IgG4 antibodies engage in Fab-arm exchange with endogenous human IgG4 in vivo. Nat Biotechnol (2009) 27:767–71. doi: 10.1038/nbt.1553 19620983

[64] Maldonado M Diaz LA Prisayanh P Yang J Qaqish BF Aoki V. Divergent specificity development of igG1 and igG4 autoantibodies in endemic pemphigus foliaceus (Fogo selvagem). ImmunoHorizons (2017) 1:71–80. doi: 10.4049/immunohorizons.1700029 28868524 PMC5577939

[65] Reinhard L Stahl RA Hoxha E. Is primary membranous nephropathy a complement mediated disease? Mol Immunol (2020) 128:195–204. doi: 10.1016/j.molimm.2020.10.017 33142137

[66] Dainichi T Chow Z Kabashima K. IgG4, complement, and the mechanisms of blister formation in pemphigus and bullous pemphigoid. J Dermatol Sci (2017) 88:265–70. doi: 10.1016/j.jdermsci.2017.07.012 28747266

[67] Perugino CA Stone JH. IgG4-related disease: an update on pathophysiology and implications for clinical care. Nat Rev Rheumatol (2020) 16:702–14. doi: 10.1038/s41584-020-0500-7 32939060

[68] Liu X Shao C Yu C Huang H Pan R Xu K. Severe asthma as the initial clinical manifestation of IgG4-related disease: a retrospective clinical study. BMC Pulmonary Med (2022) 22:141. doi: 10.1186/s12890-022-01937-9 35413899 PMC9004153

[69] Ramezanpour M Hu H Lau A Liu S De Silva A Bolt H. Increased serum IgG4 associates with asthma and tissue eosinophilia in chronic rhinosinusitis patients. Pathogens (2020) 9:828. doi: 10.3390/pathogens9100828 33050444 PMC7600683

[70] Vergoossen DLE Plomp JJ Gstöttner C Fillié-Grijpma YE Augustinus R Verpalen R. Functional monovalency amplifies the pathogenicity of anti-MuSK IgG4 in myasthenia gravis. Proc Natl Acad Sci United States America (2021) 118:e2020635118. doi: 10.1073/pnas.2020635118 PMC8020787 33753489

[71] Vergoossen DLE Augustinus R Huijbers MG. MuSK antibodies, lessons learned from poly- and monoclonality. J Autoimmun (2020) 112:102488. doi: 10.1016/j.jaut.2020.102488 32505442

[72] Rispens T Huijbers MG. The unique properties of IgG4 and its roles in health and disease. Nat Rev Immunol (2023) 23(11):763–78. doi: 10.1038/s41577-023-00871-z PMC10123589 37095254

[73] de Lange GG. Polymorphisms of human immunoglobulins: Gm, Am, Em and Km allotypes. Exp Clin immunogenetics (1989) 6:7–17. 2698222

[74] Dugoujon JM Hazout S Loirat F Mourrieras B Crouau-Roy B Sanchez-Mazas A. GM haplotype diversity of 82 populations over the world suggests a centrifugal model of human migrations. Am J Phys Anthropology: Off Publ Am Assoc Phys Anthropologists (2004) 125:175–92. doi: 10.1002/ajpa.10405 15365983

[75] Dard P Lefranc M-P Osipova L Sanchez-Mazas A. DNA sequence variability of IGHG3 alleles associated to the main G3m haplotypes in human populations. Eur J Hum Genet (2001) 9:765–72. doi: 10.1038/sj.ejhg.5200700 11781688

[76] Calonga-Solís V Malheiros D Beltrame MH Vargas L Dourado RM Issler HC. Unveiling the diversity of immunoglobulin heavy constant gamma (IGHG) gene segments in Brazilian populations reveals 28 novel alleles and evidence of gene conversion and natural selection. Front Immunol (2019) 10:1161–1. doi: 10.3389/fimmu.2019.01161 PMC6558194 31214166

[77] van Loghem E Aalberse RC Matsumoto H. A genetic marker of human IgE heavy chains, Em (1) 1. Vox sanguinis (1984) 46:195–206. 6201004 10.1111/j.1423-0410.1984.tb00075.x

[78] Lefranc MP Lefranc G. Molecular genetics of immunoglobulin allotype expression. Hum IgG subclasses: Mol Anal structure Funct Regul (1990), 43–78. doi: 10.1016/B978-0-08-037504-5.50009-0

[79] Lefranc MP Giudicelli V Duroux P Jabado-Michaloud J Folch G Aouinti S. IMGT®, the international ImMunoGeneTics information system® 25 years on. Nucleic Acids Res (2015) 43:D413–22. doi: 10.1093/nar/gku1056 PMC4383898 25378316

[80] Ford EE Tieri D Rodriguez OL Francoeur NJ Soto J Kos JT. Flairr-seq: A method for single-molecule resolution of near full-length antibody H chain repertoires. J Immunol (2023) 210:1607–19. doi: 10.4049/jimmunol.2200825 PMC10152037 37027017

[81] Richardson SI Lambson BE Crowley AR Bashirova A Scheepers C Garrett N. IgG3 enhances neutralization potency and Fc effector function of an HIV V2-specific broadly neutralizing antibody. PloS Pathog (2019) 15:e1008064–e1008064. doi: 10.1371/journal.ppat.1008064 31841557 PMC6936867

[82] Pandey JP French MAH. GM phenotypes influence the concentrations of the four subclasses of immunoglobulin G in normal human serum. Hum Immunol (1996) 51:99–102. doi: 10.1016/S0198-8859(96)00205-4 8960912

[83] Seppälä IJ Sarvas H Mäkelä O. Low concentrations of Gm allotypic subsets G3 mg and G1 mf in homozygotes and heterozygotes. J Immunol (Baltimore Md.: 1950) (1993) 151:2529–37. 8360475

[84] Hassan MS Islam KB Hammarström L Smith CI. Regulation of C gamma 3 expression. Role of switch in the allotype-associated variation of human serum IgG3 levels. J Immunol (Baltimore Md.: 1950) (1992) 148:2555–62. 1560210

[85] Pan Q Petit-Frére C Hammarström L. An allotype-associated polymorphism in the γ3 promoter determines the germ-line γ3 transcriptional rate but does not influence switching and subsequent IgG3 production. Eur J Immunol (2000) 30:2388–93. doi: 10.1002/1521-4141(2000)30:8<2388::AID-IMMU2388>3.0.CO;2-C 10940930

[86] Kratochvil S McKay PF Chung AW Kent SJ Gilmore J Shattock RJ. Immunoglobulin G1 allotype influences antibody subclass distribution in response to HIV gp140 vaccination. Front Immunol (2017) 8:1883–3. doi: 10.3389/fimmu.2017.01883 PMC5742328 29326728

[87] Atherton A Armour KL Bell S Minson AC Clark MR. The herpes simplex virus type 1 Fc receptor discriminates between IgG1 allotypes. Eur J Immunol (2000) 30:2540–7. doi: 10.1002/1521-4141(200009)30:9<2540::AID-IMMU2540>3.0.CO;2-S 11009087

[88] Moraru M Black LE Muntasell A Portero F López-Botet M Reyburn HT. NK cell and Ig interplay in defense against herpes simplex virus type 1: epistatic interaction of CD16A and IgG1 allotypes of variable affinities modulates antibody-dependent cellular cytotoxicity and susceptibility to clinical reactivation. J Immunol (2015) 195:1676–84. doi: 10.4049/jimmunol.1500872 26179905

[89] Pandey JP Olsson J Weidung B Kothera RT Johansson A Eriksson S. An Ig γ marker genotype is a strong risk factor for Alzheimer disease, independent of apolipoprotein E ϵ4 genotype. J Immunol (2020) 205:1318–22. doi: 10.4049/jimmunol.2000351 PMC8973829 32709662

[90] Jönsson G.R. Oxelius V-A Truedsson L Braconier JH Sturfelt G SjÖholm AG. Homozygosity for the IgG2 subclass allotype G2M (n) protects against severe infection in hereditary C2 deficiency. J Immunol (2006) 177:722–8. doi: 10.4049/jimmunol.177.1.722 16785571

[91] Stapleton NM Andersen JT Stemerding AM Bjarnarson SP Verheul RC Gerritsen J. Competition for FcRn-mediated transport gives rise to short half-life of human IgG3 and offers therapeutic potential. Nat Commun (2011) 2:599–9. doi: 10.1038/ncomms1608 PMC3247843 22186895

[92] Einarsdottir H Ji Y Visser R Mo C Luo G Scherjon S. H435-containing immunoglobulin G3 allotypes are transported efficiently across the human placenta: implications for alloantibody-mediated diseases of the newborn. Transfusion (2014) 54:665–71. doi: 10.1111/trf.12334 23829325

[93] Damelang T Rogerson SJ Kent SJ Chung AW. Role of IgG3 in infectious diseases. Trends Immunol (2019) 40:197–211. doi: 10.1016/j.it.2019.01.005 30745265

[94] Galbraith GM Thiers BH Pandey JP. Gm allotype associated resistance and susceptibility to alopecia areata. Clin Exp Immunol (1984) 56:149–9. PMC1535946 6424985

[95] Recke A Konitzer S Lemcke S Freitag M Sommer NM Abdelhady M. The p.Arg435His variation of IgG3 with high affinity to FcRn is associated with susceptibility for pemphigus vulgaris-analysis of four different ethnic cohorts. Front Immunol (2018) 9:1788–8. doi: 10.3389/fimmu.2018.01788 PMC6082936 30116249

[96] Pandey JP Nietert PJ Klaamas K Kurtenkov O. A genetic variant of immunoglobulin γ2 is strongly associated with immunity to mucin 1 in patients with breast cancer. Cancer Immunology Immunotherapy (2009) 58:2025–9. doi: 10.1007/s00262-009-0709-4 PMC11030933 19365631

[97] Pandey JP Namboodiri AM. Genetic variants of IgG1 antibodies and FcγRIIIa receptors influence the magnitude of antibody-dependent cell-mediated cytotoxicity against prostate cancer cells. Oncoimmunology (2014) 3:e27317–7. doi: 10.4161/onci.27317 PMC3961482 24701371

[98] Chu TH Crowley AR Backes I Chang C Tay M Broge T. Hinge length contributes to the phagocytic activity of HIV-specific IgG1 and IgG3 antibodies. PloS Pathog (2020) 16:e1008083–e1008083. doi: 10.1371/journal.ppat.1008083 32092122 PMC7058349

[99] Kyei-Baafour E Kusi KA Arthur FKN Sarkodie-Addo T Theisen M Dodoo D. Association of immunoglobulin G3 hinge region length polymorphism with cerebral malaria in Ghanaian children. J Infect Dis (2021) 225:1786–90. doi: 10.1093/infdis/jiab548 34718631

[100] Cleary KLS Chan HTC James S Glennie MJ Cragg MS. Antibody distance from the cell membrane regulates antibody effector mechanisms. J Immunol (2017) 198:3999–4011. doi: 10.4049/jimmunol.1601473 28404636 PMC5424080

[101] Natsume A In M Takamura H Nakagawa T Shimizu Y Kitajima K. Engineered antibodies of IgG1/IgG3 mixed isotype with enhanced cytotoxic activities. Cancer Res (2008) 68:3863–72. doi: 10.1158/0008-5472.CAN-07-6297 18483271

[102] Rispens T Himly M Ooievaar-De Heer P den Bleker TH Aalberse RC. Traces of pFc’in IVIG interact with human IgG Fc domains and counteract aggregation. Eur J Pharm Sci (2010) 40:62–8. doi: 10.1016/j.ejps.2010.03.001 20211252

[103] Warrender AK Kelton W. Beyond allotypes: the influence of allelic diversity in antibody constant domains. Front Immunol (2020) 11:2016–6. doi: 10.3389/fimmu.2020.02016 PMC7461860 32973808

[104] Zhang L Ding Z Heyman B. IgG3-antigen complexes are deposited on follicular dendritic cells in the presence of C1q and C3. Sci Rep (2017) 7:1–11. doi: 10.1038/s41598-017-05704-3 28710441 PMC5511153

[105] Giuntini S Granoff DM Beernink PT Ihle O Bratlie D Michaelsen TE. Human IgG1, IgG3, and IgG3 hinge-truncated mutants show different protection capabilities against meningococci depending on the target antigen and epitope specificity. Clin Vaccine Immunol (2016) 23:698–706. doi: 10.1128/CVI.00193-16 27307451 PMC4979173

[106] Rösner T Derer S Kellner C Dechant M Lohse S Vidarsson G. An IgG3 switch variant of rituximab mediates enhanced complement-dependent cytotoxicity against tumour cells with low CD20 expression levels. Br J haematology (2013) 161:282–6. doi: 10.1111/bjh.12209 23294176

[107] Damelang T de Taeye SW Rentenaar R Roya-Kouchaki K de Boer E Derksen NIL. The influence of human igG subclass and allotype on complement activation. J Immunol (2023) 211:1725–35. doi: 10.4049/jimmunol.2300307 PMC10656437 37843500

[108] Monnet C Jorieux S Urbain R Fournier N Bouayadi K De Romeuf C. Selection of IgG variants with increased FcRn binding using random and directed mutagenesis: impact on effector functions. Front Immunol (2015) 6:39–9. doi: 10.3389/fimmu.2015.00039 PMC4316771 25699055

[109] Schoch A Kettenberger H Mundigl O Winter G Engert J Heinrich J. Charge-mediated influence of the antibody variable domain on FcRn-dependent pharmacokinetics. Proc Natl Acad Sci (2015) 112:5997–6002. doi: 10.1073/pnas.1408766112 25918417 PMC4434771

[110] van Loghem E Frangione B Recht B Franklin EC Staphylococcal protein A. and human IgG subclasses and allotypes. Scandinavian J Immunol (1982) 15:275–8. doi: 10.1111/j.1365-3083.1982.tb00649.x 7089488

[111] Crowley AR Richardson SI Tuyishime M Jennewein M Bailey MJ Lee J. Functional consequences of allotypic polymorphisms in human immunoglobulin G subclasses. Immunogenetics (2023) 75:1–16. doi: 10.1007/s00251-022-01272-7 35904629 PMC9845132

[112] Redpath S Michaelsen T Sandlie I Clark MR. Activation of complement by human IgG1 and human IgG3 antibodies against the human leucocyte antigen CD52. Immunology (1998) 93:595–5. doi: 10.1046/j.1365-2567.1998.00472.x PMC1364140 9659234

[113] Schjoldager KT Narimatsu Y Joshi HJ Clausen H. Global view of human protein glycosylation pathways and functions. Nat Rev Mol Cell Biol (2020) 21:729–49. doi: 10.1038/s41580-020-00294-x 33087899

[114] Reily C Stewart TJ Renfrow MB Novak J. Glycosylation in health and disease. Nat Rev Nephrol (2019) 15:346–66. doi: 10.1038/s41581-019-0129-4 PMC6590709 30858582

[115] Oosterhoff JJ Larsen MD van der Schoot CE Vidarsson G. Afucosylated IgG responses in humans–structural clues to the regulation of humoral immunity. Trends Immunol (2022) 43(10):800–14. doi: 10.1016/j.it.2022.08.001 PMC9395167 36008258

[116] van de Bovenkamp F Derksen NIL Ooijevaar-de Heer P Van Schie KA Kruithof S Berkowska MA. Adaptive antibody diversification through N-linked glycosylation of the immunoglobulin variable region. Proc Natl Acad Sci (2018) 115:1901–6. doi: 10.1073/pnas.1711720115 PMC5828577 29432186

[117] Koers J Derksen NIL Ooijevaar-de Heer P Nota B van de Bovenkamp FS Vidarsson G. Biased N-glycosylation site distribution and acquisition across the antibody V region during B cell maturation. J Immunol (2019) 202:2220–8. doi: 10.4049/jimmunol.1801622 30850477

[118] Baković M Selman MHJ Hoffmann M Rudan I Campbell H Deelder AM. High-throughput IgG Fc N-glycosylation profiling by mass spectrometry of glycopeptides. J Proteome Res (2013) 12:821–31. doi: 10.1021/pr300887z 23298168

[119] Menni C Keser T Mangino M Bell JT Erte I Akmačić I. Glycosylation of immunoglobulin g: role of genetic and epigenetic influences. PloS One (2013) 8:e82558–8. doi: 10.1371/journal.pone.0082558 PMC3855797 24324808

[120] Trbojević Akmačić I Ventham NT Theodoratou E Vučković F Kennedy NA Krištić J. Inflammatory bowel disease associates with proinflammatory potential of the immunoglobulin G glycome. Inflammatory bowel Dis (2015) 21:1237–47. doi: 10.1097/MIB.0000000000000372 PMC4450892 25895110

[121] Wuhrer M Selman MHJ McDonnell LA Kümpfel T Derfuss T Khademi M. Pro-inflammatory pattern of IgG1 Fc glycosylation in multiple sclerosis cerebrospinal fluid. J Neuroinflamm (2015) 12:1–14. doi: 10.1186/s12974-015-0450-1 PMC4683913 26683050

[122] Dekkers G Rispens T Vidarsson G. Novel concepts of altered immunoglobulin G galactosylation in autoimmune diseases. Front Immunol (2018) 9:553–3. doi: 10.3389/fimmu.2018.00553 PMC5867308 29616041

[123] Mimura Y Church S Ghirlando R Ashton PR Dong S Goodall M. The influence of glycosylation on the thermal stability and effector function expression of human IgG1-Fc: properties of a series of truncated glycoforms. Mol Immunol (2000) 37:697–706. doi: 10.1016/S0161-5890(00)00105-X 11275255

[124] Borrok MJ Jung ST Kang TH Monzingo AF Georgiou G. Revisiting the role of glycosylation in the structure of human IgG Fc. ACS Chem Biol (2012) 7:1596–602. doi: 10.1021/cb300130k PMC3448853 22747430

[125] Subedi GP Barb AW. The structural role of antibody N-glycosylation in receptor interactions. Structure (2015) 23:1573–83. doi: 10.1016/j.str.2015.06.015 PMC4558368 26211613

[126] Lee C-H Romain G Yan W Watanabe M Charab W Todorova B. IgG Fc domains that bind C1q but not effector Fcγ receptors delineate the importance of complement-mediated effector functions. Nat Immunol (2017) 18:889–98. doi: 10.1038/ni.3770 PMC6015732 28604720

[127] Shields RL Lai J Keck R O'Connell LY Hong K Meng YG. Lack of fucose on human IgG1 N-linked oligosaccharide improves binding to human FcγRIII and antibody-dependent cellular toxicity. J Biol Chem (2002) 277:26733–40. doi: 10.1074/jbc.M202069200 11986321

[128] Dekkers G Treffers L Plomp R Bentlage AEH de Boer M Koeleman CAM. Decoding the human immunoglobulin G-glycan repertoire reveals a spectrum of Fc-receptor-and complement-mediated-effector activities. Front Immunol (2017) 8:877–7. doi: 10.3389/fimmu.2017.00877 PMC5539844 28824618

[129] Quast I Keller CW Maurer MA Giddens JP Tackenberg B Wang L-X. Sialylation of IgG Fc domain impairs complement-dependent cytotoxicity. J Clin Invest (2015) 125:4160–70. doi: 10.1172/JCI82695 PMC4639970 26436649

[130] Niwa R Natsume A Uehara A Wakitani M Iida S Uchida K. IgG subclass-independent improvement of antibody-dependent cellular cytotoxicity by fucose removal from Asn297-linked oligosaccharides. J Immunol Methods (2005) 306:151–60. doi: 10.1016/j.jim.2005.08.009 16219319

[131] Chung AW Crispin M Pritchard L Robinson H Gorny MK Yu X. Identification of antibody glycosylation structures that predict monoclonal antibody Fc-effector function. AIDS (London England) (2014) 28:2523–3. doi: 10.1097/QAD.0000000000000444 PMC4429604 25160934

[132] Lin C-W Tsai M-H Li S-T Tsai T-I Chu K-C Liu Y-C. A common glycan structure on immunoglobulin G for enhancement of effector functions. Proc Natl Acad Sci (2015) 112:10611–6. doi: 10.1073/pnas.1513456112 PMC4553773 26253764

[133] van Osch TLJ Nouta J Derksen NIL van Mierlo G van der Schoot CE Wuhrer M. Fc galactosylation promotes hexamerization of human IgG1, leading to enhanced classical complement activation. J Immunol (2021) 207:1545–54. doi: 10.4049/jimmunol.2100399 PMC8428746 34408013

[134] Kao D Danzer H Collin M Groß A Eichler J Stambuk J. A monosaccharide residue is sufficient to maintain mouse and human igG subclass activity and directs igG effector functions to cellular fc receptors. Cell Rep (2015) 13:2376–85. doi: 10.1016/j.celrep.2015.11.027 26670049

[135] Falconer DJ Subedi GP Marcella AM Barb AW. Antibody fucosylation lowers the FcγRIIIa/CD16a affinity by limiting the conformations sampled by the N162-glycan. ACS Chem Biol (2018) 13:2179–89. doi: 10.1021/acschembio.8b00342 PMC6415948 30016589

[136] Shinkawa T Nakamura K Yamane N Shoji-Hosaka E Kanda Y Sakurada M. The absence of fucose but not the presence of galactose or bisecting N-acetylglucosamine of human IgG1 complex-type oligosaccharides shows the critical role of enhancing antibody-dependent cellular cytotoxicity. J Biol Chem (2003) 278:3466–73. doi: 10.1074/jbc.M210665200 12427744

[137] Ferrara C Grau S Jäger C Sondermann P Brünker P Waldhauer I. Unique carbohydrate–carbohydrate interactions are required for high affinity binding between FcγRIII and antibodies lacking core fucose. Proc Natl Acad Sci (2011) 108:12669–74. doi: 10.1073/pnas.1108455108 PMC3150898 21768335

[138] Temming AR de Taeye SW de Graaf EL de Neef LA Dekkers G Bruggeman CW. Functional attributes of antibodies, effector cells, and target cells affecting NK cell–mediated antibody-dependent cellular cytotoxicity. J Immunol (2019) 203:3126–35. doi: 10.4049/jimmunol.1900985 31748349

[139] Karampatzakis A Brož P Rey C Önfelt B Cruz De Matos GDS Rycroft D. Antibody afucosylation augments CD16-mediated serial killing and IFNγ secretion by human natural killer cells. Front Immunol (2021) 12:641521–1. doi: 10.3389/fimmu.2021.641521 PMC8008054 33796107

[140] Wuhrer M Porcelijn L Kapur R Koeleman CAM Deelder AM De Haas M. Regulated glycosylation patterns of IgG during alloimmune responses against human platelet antigens. J Proteome Res (2009) 8:450–6. doi: 10.1021/pr800651j 18942870

[141] Ackerman ME Crispin M Yu X Baruah K Boesch AW Harvey DJ. Natural variation in Fc glycosylation of HIV-specific antibodies impacts antiviral activity. J Clin Invest (2013) 123:2183–92. doi: 10.1172/JCI65708 PMC3637034 23563315

[142] Kapur R Kustiawan I Vestrheim A Koeleman CAM Visser R Einarsdottir HK. A prominent lack of IgG1-Fc fucosylation of platelet alloantibodies in pregnancy. Blood (2014) 123:471–80. doi: 10.1182/blood-2013-09-527978 PMC3901064 24243971

[143] Kapur R Della Valle L Sonneveld M Hipgrave Ederveen A Visser R Ligthart P. Low anti-RhD IgG-Fc-fucosylation in pregnancy: a new variable predicting severity in haemolytic disease of the fetus and newborn. Br J Haematology (2014) 166:936–45. doi: 10.1111/bjh.12965 PMC4282073 24909983

[144] Auton A Abecasis GR Altshuler DM Durbin RM Abecasis GR Bentley DR. A global reference for human genetic variation. Nature (2015) 526:68–74. doi: 10.1038/nature15393 26432245 PMC4750478

[145] Larsen MD Lopez-Perez M Dickson EK Ampomah P Tuikue Ndam N Nouta J. Afucosylated Plasmodium falciparum-specific IgG is induced by infection but not by subunit vaccination. Nat Commun (2021) 12:5838–8. doi: 10.1038/s41467-021-26118-w PMC8492741 34611164

[146] Larsen MD de Graaf EL Sonneveld ME Plomp HR Nouta J Hoepel W. Afucosylated IgG characterizes enveloped viral responses and correlates with COVID-19 severity. Science (2021) 371:eabc8378–eabc8378. doi: 10.1126/science.abc8378 33361116 PMC7919849

[147] Gardinassi LG Dotz V Hipgrave Ederveen A de Almeida RP Nery Costa CH Costa DL. Clinical severity of visceral leishmaniasis is associated with changes in immunoglobulin g fc N-glycosylation. MBio (2014) 5:e01844–14. doi: 10.1128/mBio.01844-14 PMC4324239 25467439

[148] Selman MHJ de Jong SE Soonawala D Kroon FP Adegnika AA Deelder AM. Changes in antigen-specific IgG1 Fc N-glycosylation upon influenza and tetanus vaccination. Mol Cell Proteomics (2012) 11:M111.014563. doi: 10.1074/mcp.M111.014563 PMC3322571 22184099

[149] Mahan AE Jennewein MF Suscovich T Dionne K Tedesco J Chung AW. Antigen-specific antibody glycosylation is regulated *via* vaccination. PloS Pathog (2016) 12:e1005456–e1005456. doi: 10.1371/journal.ppat.1005456 26982805 PMC4794126

[150] Van Coillie J Pongracz T Rahmöller J Chen H-J Geyer CE van Vught LA. The BNT162b2 mRNA SARS-CoV-2 vaccine induces transient afucosylated IgG1 in naive but not in antigen-experienced vaccinees. EBioMedicine (2023) 87:104408–8. doi: 10.1016/j.ebiom.2022.104408 PMC9756879 36529104

[151] Wang TT Maamary J Tan GS Bournazos S Davis CW Krammer F. Anti-HA glycoforms drive B cell affinity selection and determine influenza vaccine efficacy. Cell (2015) 162:160–9. doi: 10.1016/j.cell.2015.06.026 PMC4594835 26140596

[152] Lippold S Nicolardi S Domínguez-Vega E Heidenreich A-K Vidarsson G Reusch D. Glycoform-resolved FcγRIIIa affinity chromatography–mass spectrometry. MAbs Taylor Francis (2019) 11(7):1191–6. doi: 10.1080/19420862.2019.1636602 PMC6748599 31276431

[153] Jennewein MF Alter G. The immunoregulatory roles of antibody glycosylation. Trends Immunol (2017) 38:358–72. doi: 10.1016/j.it.2017.02.004 28385520

[154] Scallon BJ Tam SH McCarthy SG Cai AN Raju TS. Higher levels of sialylated Fc glycans in immunoglobulin G molecules can adversely impact functionality. Mol Immunol (2007) 44:1524–34. doi: 10.1016/j.molimm.2006.09.005 17045339

[155] Kaneko Y Nimmerjahn F Ravetch JV. Anti-inflammatory activity of immunoglobulin G resulting from Fc sialylation. Science (2006) 313:670–3. doi: 10.1126/science.1129594 16888140

[156] Schwab I Mihai S Seeling M Kasperkiewicz M Ludwig RJ Nimmerjahn F. Broad requirement for terminal sialic acid residues and FcγRIIB for the preventive and therapeutic activity of intravenous immunoglobulins in vivo. Eur J Immunol (2014) 44:1444–53. doi: 10.1002/eji.201344230 24505033

[157] Schwab I Seeling M Biburger M Aschermann S Nitschke L Nimmerjahn F. B cells and CD 22 are dispensable for the immediate antiinflammatory activity of intravenous immunoglobulins in vivo. Eur J Immunol (2012) 42:3302–9. doi: 10.1002/eji.201242710 22945870

[158] Peschke B Keller CW Weber P Quast I Lünemann JD. Fc-galactosylation of human immunoglobulin gamma isotypes improves C1q binding and enhances complement-dependent cytotoxicity. Front Immunol (2017) 8:646–6. doi: 10.3389/fimmu.2017.00646 PMC5459932 28634480

[159] van Osch TLJ Oosterhoff JJ Bentlage AEH Nouta J Koeleman CAM Geerdes DM. Fc galactosylation of anti-platelet hIgG1 alloantibodies enhance complement activation on platelets. Haematologica (2022) 107(10):2432–44. doi: 10.3324/haematol.2021.280493 PMC9521249 35354253

[160] Wei B Gao X Cadang L Izadi S Liu P Zhang H-M. Fc galactosylation follows consecutive reaction kinetics and enhances immunoglobulin G hexamerization for complement activation. MAbs Taylor Francis (2021) 13(1):1893427–1893427. doi: 10.1080/19420862.2021.1893427 PMC7946005 33682619

[161] Lubbers R Oostindie SC Dijkstra DJ Parren PWHI Verheul MK Abendstein L. Carbamylation reduces the capacity of IgG for hexamerization and complement activation. Clin Exp Immunol (2020) 200:1–11. doi: 10.1111/cei.13411 31853959 PMC7066385

[162] van den Bremer ETJ Beurskens FJ Voorhorst M Engelberts PJ de Jong RN van der Boom BG. Human IgG is produced in a pro-form that requires clipping of C-terminal lysines for maximal complement activation. MAbs Taylor Francis (2015) 7(4):672–80. doi: 10.1080/19420862.2015.1046665 PMC4622059 26037225

[163] de Haan N Falck D Wuhrer M. Monitoring of immunoglobulin N-and O-glycosylation in health and disease. Glycobiology (2020) 30:226–40. doi: 10.1093/glycob/cwz048 PMC7225405 31281930

[164] van de Bovenkamp FS Derksen NIL Van Breemen MJ De Taeye SW Ooijevaar-de Heer P Sanders RW. Variable domain N-linked glycans acquired during antigen-specific immune responses can contribute to immunoglobulin G antibody stability. Front Immunol (2018) 9:740–0. doi: 10.3389/fimmu.2018.00740 PMC5906590 29706962

[165] Hafkenscheid L Bondt A Scherer HU Huizinga TWJ Wuhrer M Toes REM. Structural analysis of variable domain glycosylation of anti-citrullinated protein antibodies in rheumatoid arthritis reveals the presence of highly sialylated glycans. Mol Cell Proteomics (2017) 16:278–87. doi: 10.1074/mcp.M116.062919 PMC5294214 27956708

[166] Bondt A Wuhrer M Kuijper TM Hazes JMW Dolhain RJEM. Fab glycosylation of immunoglobulin G does not associate with improvement of rheumatoid arthritis during pregnancy. Arthritis Res Ther (2016) 18:1–6. doi: 10.1186/s13075-016-1172-1 27887659 PMC5123206

[167] Rombouts Y Willemze A van Beers JJBC Shi J Kerkman PF van Toorn L. Extensive glycosylation of ACPA-IgG variable domains modulates binding to citrullinated antigens in rheumatoid arthritis. Ann rheumatic Dis (2016) 75:578–85. doi: 10.1136/annrheumdis-2014-206598 25587188

[168] Koers J Sciarrillo R Derksen NIL Vletter EM Fillié-Grijpma YE Raveling-Eelsing E. Differences in IgG autoantibody Fab glycosylation across autoimmune diseases. J Allergy Clin Immunol (2023) 151(6):1646–54. doi: 10.1016/j.jaci.2022.10.035 36716825

[169] Culver EL van de Bovenkamp FS Derksen NIL Koers J Cargill T Barnes E. Unique patterns of glycosylation in immunoglobulin subclass G4-related disease and primary sclerosing cholangitis. J Gastroenterol Hepatol (2019) 34:1878–86. doi: 10.1111/jgh.14512 PMC6899843 30345709

[170] Vletter EM Koning MT Scherer HU Veelken H Toes REM. A comparison of immunoglobulin variable region N-linked glycosylation in healthy donors, autoimmune disease and lymphoma. Front Immunol (2020) 11:241–1. doi: 10.3389/fimmu.2020.00241 PMC7040075 32133009

[171] Brinkhaus M Pannecoucke E van der Kooi EJ Bentlage AEH Derksen NIL Andries J. The Fab region of IgG impairs the internalization pathway of FcRn upon Fc engagement. Nat Commun (2022) 13:6073. doi: 10.1038/s41467-022-33764-1 36241613 PMC9568614

[172] Volkov M Brinkhaus M van Schie KA Bondt A Kissel T van der Kooi EJ. IgG fab glycans hinder fcRn-mediated placental transport. J Immunol (2023) 210:158–67. doi: 10.4049/jimmunol.2200438 36480251

[173] Kissel T Ge C Hafkenscheid L Kwekkeboom JC Slot LM Cavallari M. Surface Ig variable domain glycosylation affects autoantigen binding and acts as threshold for human autoreactive B cell activation. Sci Adv (2022) 8:eabm1759. doi: 10.1126/sciadv.abm1759 35138894 PMC8827743

[174] Lardinoisid OM Deterding LJ Hess JJ Poulton CJ Henderson CD Jennette JC. Immunoglobulins G from patients with ANCA-associated vasculitis are atypically glycosylated in both the Fc and Fab regions and the relation to disease activity. PloS One (2019) 14(2):e0213215. doi: 10.1371/journal.pone.0213215 30818380 PMC6395067

[175] Plomp R Dekkers G Rombouts Y Visser R Koeleman CAM Kammeijer GSM. Hinge-region O-glycosylation of human immunoglobulin G3 (IgG3)*[S]. Mol Cell Proteomics (2015) 14:1373–84. doi: 10.1074/mcp.M114.047381 PMC4424406 25759508

[176] Julenius K Mølgaard A Gupta R Brunak S. Prediction, conservation analysis, and structural characterization of mammalian mucin-type O-glycosylation sites. Glycobiology (2005) 15:153–64. doi: 10.1093/glycob/cwh151 15385431

[177] Richards JO Karki S Lazar GA Chen H Dang W Desjarlais JR. Optimization of antibody binding to FcγRIIa enhances macrophage phagocytosis of tumor cells. Mol Cancer Ther (2008) 7:2517–27. doi: 10.1158/1535-7163.MCT-08-0201 18723496

[178] Mimoto F Katada H Kadono S Igawa T Kuramochi T Muraoka M. Engineered antibody Fc variant with selectively enhanced FcγRIIb binding over both FcγRIIaR131 and FcγRIIaH131. Protein Engineering Design Selection (2013) 26:589–98. doi: 10.1093/protein/gzt022 PMC3785249 23744091

[179] Shields RL Namenuk AK Hong K Meng YG Rae J Briggs J. High resolution mapping of the binding site on human IgG1 for FcγRI, FcγRII, FcγRIII, and FcRn and design of IgG1 variants with improved binding to the FcγR. J Biol Chem (2001) 276:6591–604. doi: 10.1074/jbc.M009483200 11096108

[180] Petkova SB Akilesh S Sproule TJ Christianson GJ Al Khabbaz H Brown AC. Enhanced half-life of genetically engineered human IgG1 antibodies in a humanized FcRn mouse model: potential application in humorally mediated autoimmune disease. Int Immunol (2006) 18(12):1759–69. doi: 10.1093/intimm/dxl110 17077181

[181] Moore GL Chen H Karki S Lazar GA. Engineered Fc variant antibodies with enhanced ability to recruit complement and mediate effector functions. MAbs Taylor Francis (2010), 181–9. doi: 10.4161/mabs.2.2.11158 PMC2840237 20150767

[182] Smith P DiLillo DJ Bournazos S Li F Ravetch JV. Mouse model recapitulating human Fcγ receptor structural and functional diversity. Proc Natl Acad Sci (2012) 109:6181–6. doi: 10.1073/pnas.1203954109 PMC3341029 22474370

[183] Idusogie EE Presta LG Gazzano-Santoro H Totpal K Wong PY Ultsch M. Mapping of the C1q binding site on rituxan, a chimeric antibody with a human IgG1 Fc. J Immunol (2000) 164(8):4178–84. doi: 10.4049/jimmunol.164.8.4178 10754313

[184] Moldt B Schultz N Dunlop DC Alpert MD Harvey JD Evans DT. A panel of IgG1 b12 variants with selectively diminished or enhanced affinity for fcγ receptors to define the role of effector functions in protection against HIV. J Virol (2011) 85(20):10572–81. doi: 10.1128/JVI.05541-11 PMC3187489 21849450

[185] Tao MH Morrison SL. Studies of aglycosylated chimeric mouse-human IgG. role of carbohydrate in the structure and effector functions mediated by the human IgG constant region. J Immunol (1989) 143(8):2595–601. 2507634

[186] Idusogie EE Wong PY Presta LG Gazzano-Santoro H Totpal K Ultsch M. Engineered antibodies with increased activity to recruit complement. J Immunol (2001) 166:2571–5. doi: 10.4049/jimmunol.166.4.2571 11160318

[187] Canfield SM Morrison SL. The binding affinity of human IgG for its high affinity fc receptor is determined by multiple amino acids in the CH2 domain and is modulated by the hinge region. J Exp Med (1991) 173(6):1483–91. doi: 10.1084/jem.173.6.1483 PMC2190830 1827828

[188] de Jong RN Beurskens FJ Verploegen S Strumane K van Kampen MD Voorhorst M. A novel platform for the potentiation of therapeutic antibodies based on antigen-dependent formation of IgG hexamers at the cell surface. PloS Biol (2016) 14:e1002344–e1002344. doi: 10.1371/journal.pbio.1002344 26736041 PMC4703389

[189] Yeung YA Leabman MK Marvin JS Qiu J Adams CW Lien S. Engineering human IgG1 affinity to human neonatal Fc receptor: impact of affinity improvement on pharmacokinetics in primates. J Immunol (2009) 182(12):7663–71. doi: 10.4049/jimmunol.0804182 19494290

[190] Dall'Acqua WF Cook KE Damschroder MM Woods RM Wu H. Modulation of the effector functions of a human IgG1 through engineering of its hinge region. J Immunol (2006) 177(2):1129–38. doi: 10.4049/jimmunol.177.2.1129 16818770

[191] Schlothauer T Herter S Koller CF Grau-Richards S Steinhart V Spick C. Novel human IgG1 and IgG4 Fc-engineered antibodies with completely abolished immune effector functions. Protein Engineering Design Selection (2016) 29:457–66. doi: 10.1093/protein/gzw040 27578889

[192] Xu D Alegre M-L Varga SS Rothermel AL Collins AM Pulito VL. *In vitro* characterization of five humanized OKT3 effector function variant antibodies. Cell Immunol (2000) 200:16–26. doi: 10.1006/cimm.2000.1617 10716879

[193] Hezareh M Hessell AJ Jensen RC van de Winkel JGJ Parren PW. Effector function activities of a panel of mutants of a broadly neutralizing antibody against human immunodeficiency virus type 1. J Virol (2001) 75:12161–8. doi: 10.1128/JVI.75.24.12161-12168.2001 PMC116112 11711607

[194] Vafa O Gilliland GL Brezski RJ Strake B Wilkinson T Lacy ER. An engineered Fc variant of an IgG eliminates all immune effector functions *via* structural perturbations. Methods (San Diego Calif.) (2014) 65:114–26. 10.1016/j.ymeth.2013.06.035 23872058

[195] Hori Y Ohmine K Katada H Noguchi Y Sato K Nambu T. Elimination of plasma soluble antigen in cynomolgus monkeys by combining pH-dependent antigen binding and novel Fc engineering. Mabs Taylor Francis (2022), 2068213–2068213. doi: 10.1080/19420862.2022.2068213 PMC9067469 35482905

[196] Chu SY Vostiar I Karki S Moore GL Lazar GA Pong E. Inhibition of B cell receptor-mediated activation of primary human B cells by coengagement of CD19 and FcγRIIb with Fc-engineered antibodies. Mol Immunol (2008) 45:3926–33. doi: 10.1016/j.molimm.2008.06.027 18691763

[197] Horton HM Bernett MJ Pong E Peipp M Karki S Chu SY. Potent *in vitro* and *in vivo* activity of an fc-engineered anti-CD19 monoclonal antibody against lymphoma and leukemia. Cancer Res (2008) 68(19):8049–57. doi: 10.1158/0008-5472.CAN-08-2268 18829563

[198] Lazar GA Dang W Karki S Vafa O Peng JS Hyun L. Engineered antibody Fc variants with enhanced effector function. Proc Natl Acad Sci (2006) 103:4005–10. doi: 10.1073/pnas.0508123103 PMC1389705 16537476

[199] Cheney CM Stephens DM Mo X Rafiq S Butchar J Flynn JM. Ocaratuzumab, an fc-engineered antibody demonstrates enhanced antibody-dependent cell-mediated cytotoxicity in chronic lymphocytic leukemia. MAbs (2014) 6(3):749–55. doi: 10.4161/mabs.28282 PMC4011919 24594909

[200] Hinton PR Xiong JM Johlfs MG Tang MT Keller S Tsurushita N. An engineered human IgG1 antibody with longer serum half-life. J Immunol (2006) 176(1):346–56. doi: 10.4049/jimmunol.176.1.346 16365427

[201] Mackness BC Jaworski JA Boudanova E Park A Valente D Mauriac C. Antibody Fc engineering for enhanced neonatal Fc receptor binding and prolonged circulation half-life. MAbs Taylor Francis (2019) pp:1276–88. doi: 10.1080/19420862.2019.1633883 PMC6748615 31216930

[202] Datta-Mannan A Witcher DR Tang Y Watkins J Wroblewski VJ. Monoclonal antibody clearance. impact of modulating the interaction of IgG with the neonatal fc receptor. J Biol Chem (2007) 282(3):1709–17. doi: 10.1074/jbc.M607161200 17135257

[203] Sazinsky SL Ott RG Silver NW Tidor B Ravetch JV Wittrup KD. Aglycosylated immunoglobulin G1 variants productively engage activating Fc receptors. Proc Natl Acad Sci (2008) 105:20167–72. doi: 10.1073/pnas.0809257105 PMC2629253 19074274

[204] Zalevsky J Chamberlain AK Horton HM Karki S Leung IW Sproule TJ. Enhanced antibody half-life improves *in vivo* activity. Nat Biotechnol (2010) 28(2):157–9. doi: 10.1038/nbt.1601 PMC2855492 20081867

[205] Vaccaro C Bawdon R Wanjie S Ober RJ Ward ES. Divergent activities of an engineered antibody in murine and human systems have implications for therapeutic antibodies. Proc Natl Acad Sci (2006) 103:18709–14. doi: 10.1073/pnas.0606304103 PMC1693727 17116867

[206] Oganesyan V Gao C Shirinian L Wu H Dall'Acqua WF. Structural characterization of a human Fc fragment engineered for lack of effector functions. Acta Crystallographica Section D: Biol Crystallogr (2008) 64:700–4. doi: 10.1107/S0907444908007877 PMC2467532 18560159

[207] Borrok MJ Mody N Lu X Kuhn ML Wu H Dall'Acqua WF. An "Fc-silenced" IgG1 format with extended half-life designed for improved stability. J Pharm Sci (2017) 106(4):1008–17. doi: 10.1016/j.xphs.2016.12.023 28057542

[208] Wilkinson I Anderson S Fry J Julien LA Neville D Qureshi O. Fc-engineered antibodies with immune effector functions completely abolished. PloS One (2021) 16:e0260954–e0260954. doi: 10.1371/journal.pone.0260954 34932587 PMC8691596

[209] Engelberts PJ Hiemstra IH de Jong B Schuurhuis DH Meesters J Hernandez IB. DuoBody-CD3xCD20 induces potent T-cell-mediated killing of Malignant B cells in preclinical models and provides opportunities for subcutaneous dosing. EBioMedicine (2020) 52:102625–5. doi: 10.1016/j.ebiom.2019.102625 PMC6992935 31981978

[210] Mimoto F Igawa T Kuramochi T Katada H Kadono S Kamikawa T. Novel asymmetrically engineered antibody Fc variant with superior FcγR binding affinity and specificity compared with afucosylated Fc variant. MAbs Taylor Francis (2013) pp:229–36. doi: 10.4161/mabs.23452 PMC3893233 23406628

[211] Liu R Oldham RJ Teal E Beers SA Cragg MS. Fc-engineering for modulated effector functions—improving antibodies for cancer treatment. Antibodies (2020) 9(4):64–4. doi: 10.3390/antib9040064 PMC7709126 33212886

[212] Ravetch JV Bournazos S. Human IgG fc domain variants with improved effector function. U.S. Patent Application No. 16/424,639.

[213] Gunn BM Lu R Slein MD Ilinykh PA Huang K Atyeo C. A fc engineering approach to define functional humoral correlates of immunity against ebola virus. Immunity (2021) 54(4):815–28.e5. doi: 10.1016/j.immuni.2021.03.009 33852832 PMC8111768

[214] Dall'Acqua WF Woods RM Ward ES Palaszynski SR Patel NK Brewah YA. Increasing the affinity of a human IgG1 for the neonatal Fc receptor: biological consequences. J Immunol (2002) 169:5171–80. doi: 10.4049/jimmunol.169.9.5171 12391234

[215] Dall'Acqua WF Kiener PA Wu H. Properties of human IgG1s engineered for enhanced binding to the neonatal Fc receptor (FcRn). J Biol Chem (2006) 281:23514–24. doi: 10.1074/jbc.M604292200 16793771

[216] Qiao S-W Kobayashi K Johansen F-E Sollid LM Andersen JT Milford E. Dependence of antibody-mediated presentation of antigen on FcRn. Proc Natl Acad Sci (2008) 105:9337–42. doi: 10.1073/pnas.0801717105 PMC2453734 18599440

[217] Monnet C Jorieux S Souyris N Zaki O Jacquet A Fournier N. Combined glyco-and protein-Fc engineering simultaneously enhance cytotoxicity and half-life of a therapeutic antibody. MAbs Taylor Francis (2014), 422–36. doi: 10.4161/mabs.27854 PMC3984331 24492301

[218] Bas M Terrier A Jacque E Dehenne A Pochet-Béghin V Beghin C. Fc sialylation prolongs serum half-life of therapeutic antibodies. J Immunol (2019) 202:1582–94. doi: 10.4049/jimmunol.1800896 30683704

[219] Booth BJ Ramakrishnan B Narayan K Wollacott AM Babcock GJ Shriver Z. Extending human IgG half-life using structure-guided design. MAbs Taylor Francis (2018), 1098–110. doi: 10.1080/19420862.2018.1490119 PMC6204840 29947573

[220] Horton HM Chu SY Ortiz EC Pong E Cemerski S Leung IW. Antibody-mediated coengagement of FcγRIIb and b cell receptor complex suppresses humoral immunity in systemic lupus erythematosus. J Immunol (2011) 186(7):4223–33. doi: 10.4049/jimmunol.1003412 21357255

[221] Lee C-H Kang TH Godon O Watanabe M Delidakis G Gillis CM. An engineered human Fc domain that behaves like a pH-toggle switch for ultra-long circulation persistence. Nat Commun (2019) 10:5031–1. doi: 10.1038/s41467-019-13108-2 PMC6834678 31695028

[222] Bournazos S Klein F Pietzsch J Seaman MS Nussenzweig MC Ravetch JV. Broadly neutralizing anti-HIV-1 antibodies require fc effector functions for *in vivo* activity. Cell (2014) 158(6):1243–53. doi: 10.1016/j.cell.2014.08.023 PMC4167398 25215485

[223] Ahmed AA Keremane SR Vielmetter J Bjorkman PJ. Structural characterization of GASDALIE Fc bound to the activating Fc receptor FcγRIIIa. J Struct Biol (2016) 194:78–89. doi: 10.1016/j.jsb.2016.02.001 26850169 PMC4769027

[224] An Z Forrest G Moore R Cukan M Haytko P Huang L. IgG2m4, an engineered antibody isotype with reduced fc function. MAbs (2009) 1(6):572–9. doi: 10.4161/mabs.1.6.10185 PMC2791314 20073128

[225] Moore GL Bernett MJ Rashid R Pong EW Nguyen D-HT Jacinto J. A robust heterodimeric Fc platform engineered for efficient development of bispecific antibodies of multiple formats. Methods (2019) 154:38–50. doi: 10.1016/j.ymeth.2018.10.006 30366098

[226] Stavenhagen JB Gorlatov S Tuaillon N Rankin CT Li H Burke S. Fc optimization of therapeutic antibodies enhances their ability to kill tumor cells in *vitro* and controls tumor expansion in *vivo via* low-affinity activating Fcγ receptors. Cancer Res (2007) 67:8882–90. doi: 10.1158/0008-5472.CAN-07-0696 17875730

[227] Nordstrom JL Gorlatov S Zhang W Yang Y Huang L Burke S. Anti-tumor activity and toxicokinetics analysis of MGAH22, an anti-HER2 monoclonal antibody with enhanced Fcγ receptor binding properties. Breast Cancer Res (2011) 13:1–14. doi: 10.1186/bcr3069 PMC3326565 22129105

[228] Yamane-Ohnuki N Kinoshita S Inoue-Urakubo M Kusunoki M Iida S Nakano R. Establishment of FUT8 knockout Chinese hamster ovary cells: an ideal host cell line for producing completely defucosylated antibodies with enhanced antibody-dependent cellular cytotoxicity. Biotechnol Bioeng (2004) 87(5):614–22. doi: 10.1002/bit.20151 15352059

[229] Bang YJ Giaccone G Im SA Oh DY Bauer TM Nordstrom JL. First-in-human phase 1 study of margetuximab (MGAH22), an Fc-modified chimeric monoclonal antibody, in patients with HER2-positive advanced solid tumors. Ann Oncol (2017) 28:855–61. doi: 10.1093/annonc/mdx002 PMC6246722 28119295

[230] Monnet C Jacque E de Romeuf C Fontayne A Abache T Fournier N. The dual targeting of FcRn and FcγRs *via* monomeric fc fragments results in strong inhibition of IgG-dependent autoimmune pathologies. Front Immunol (2021) 12:728322. doi: 10.3389/fimmu.2021.728322 34512662 PMC8427755

[231] Tam SH McCarthy SG Armstrong AA Somani S Wu SJ Liu X. Functional, biophysical, and structural characterization of human IgG1 and IgG4 fc variants with ablated immune functionality. Antibodies (Basel Switzerland) (2017) 6(3):12. doi: 10.3390/antib6030012 31548527 PMC6698826

[232] Stapleton NM Armstrong-Fisher SS Andersen JT van der Schoot CE Porter C Page KR. Human IgG lacking effector functions demonstrate lower FcRn-binding and reduced transplacental transport. Mol Immunol (2018) 95:1–9. doi: 10.1016/j.molimm.2018.01.006 29367080

[233] Thommesen JE Michaelsen TE Løset G.Å. Sandlie I Brekke OH. Lysine 322 in the human IgG3 CH2 domain is crucial for antibody dependent complement activation. Mol Immunol (2000) 37:995–1004. doi: 10.1016/S0161-5890(01)00010-4 11395138

[234] Michaelsen TE Thommesen JE Ihle O Gregers TF Sandin RH Brekke OH. A mutant human IgG molecule with only one C1q binding site can activate complement and induce lysis of target cells. Eur J Immunol (2006) 36:129–38. doi: 10.1002/eji.200535178 16323243

[235] Zhang D Goldberg MV Chiu ML. Fc engineering approaches to enhance the agonism and effector functions of an anti-OX40 antibody. J Biol Chem (2016) 291:27134–46. doi: 10.1074/jbc.M116.757773 PMC5207143 27856634

[236] Zhang D Armstrong AA Tam SH McCarthy SG Luo J Gilliland GL. Functional optimization of agonistic antibodies to OX40 receptor with novel Fc mutations to promote antibody multimerization. MAbs Taylor Francis (2017), 1129–42. doi: 10.1080/19420862.2017.1358838 PMC5627589 28758875

[237] Tammen A Derer S Schwanbeck R Rösner T Kretschmer A Beurskens FJ. Monoclonal antibodies against epidermal growth factor receptor acquire an ability to kill tumor cells through complement activation by mutations that selectively facilitate the hexamerization of IgG on opsonized cells. J Immunol (2017) 198:1585–94. doi: 10.4049/jimmunol.1601268 28062698

[238] Gulati S Beurskens FJ de Kreuk B-J Roza M Zheng B DeOliveira RB. Complement alone drives efficacy of a chimeric antigonococcal monoclonal antibody. PloS Biol (2019) 17:e3000323–e3000323. doi: 10.1371/journal.pbio.3000323 31216278 PMC6602280

[239] Oostindie SC van der Horst HJ Lindorfer MA Cook EM Tupitza JC Zent CS. CD20 and CD37 antibodies synergize to activate complement by Fc-mediated clustering. haematologica (2019) 104:1841–1. doi: 10.3324/haematol.2018.207266 PMC6717598 30792198

[240] Oostindie SC van der Horst HJ Kil LP Strumane K Overdijk MB van den Brink EN. DuoHexaBody-CD37®, a novel biparatopic CD37 antibody with enhanced Fc-mediated hexamerization as a potential therapy for B-cell Malignancies. Blood Cancer J (2020) 10:30–0. doi: 10.1038/s41408-020-0292-7 PMC7186228 32341336

[241] Oostindie SC Rinaldi DA Zom GG Wester MJ Paulet D Al-Tamimi K. Logic-gated antibody pairs that selectively act on cells co-expressing two antigens. Nat Biotechnol (2022) 40:1509–19. doi: 10.1038/s41587-022-01384-1 PMC9546771 35879362

[242] van Kampen MD Kuipers-De Wilt LHAM van Egmond ML Reinders-Blankert P van den Bremer ETJ Wang G. Biophysical characterization and stability of modified IgG1 antibodies with different hexamerization propensities. J Pharm Sci (2022) 111:1587–98. doi: 10.1016/j.xphs.2022.02.016 35235843

[243] Morgan A Jones ND Nesbitt AM Chaplin L Bodmer MW Emtage JS. The N-terminal end of the CH2 domain of chimeric human IgG1 anti-HLA-DR is necessary for C1q, Fc gamma RI and Fc gamma RIII binding. Immunology (1995) 86:319–9. PMC1384012 7490135

[244] Iwayanagi Y Igawa T Maeda A Haraya K Wada NA Shibahara N. Inhibitory fcγRIIb-mediated soluble antigen clearance from plasma by a pH-dependent antigen-binding antibody and its enhancement by fc engineering. J Immunol (Baltimore Md. (2015) 1950) 195:3198–205. 10.4049/jimmunol.1401470 PMC4574519 26320252

[245] Muramatsu H Kuramochi T Katada H Ueyama A Ruike Y Ohmine K. Novel myostatin-specific antibody enhances muscle strength in muscle disease models. Sci Rep (2021) 11:2160–0. doi: 10.1038/s41598-021-81669-8 PMC7835227 33495503

[246] Stavenhagen JB Gorlatov S Tuaillon N Rankin CT Li H Burke S. Enhancing the potency of therapeutic monoclonal antibodies *via* Fc optimization. Adv Enzyme Regul (2008) 48:152–64. doi: 10.1016/j.advenzreg.2007.11.011 18177741

[247] Alegre M-L Collins AM Pulito VL Brosius RA Olson WC Zivin RA. Effect of a single amino acid mutation on the activating and immunosuppressive properties of a" humanized" OKT3 monoclonal antibody. J Immunol (Baltimore Md.: 1950) (1992) 148:3461–8. 1534096

[248] Wines BD Powell MS Parren PWHI Barnes N Hogarth PM. The IgG Fc contains distinct Fc receptor (FcR) binding sites: the leukocyte receptors FcγRI and FcγRIIa bind to a region in the Fc distinct from that recognized by neonatal FcR and protein A. J Immunol (2000) 164:5313–8. doi: 10.4049/jimmunol.164.10.5313 10799893

[249] Abdeldaim DT Schindowski K. Fc-engineered therapeutic antibodies: recent advances and future directions. Pharmaceutics (2023) 15:2402. doi: 10.3390/pharmaceutics15102402 37896162 PMC10610324

[250] Lo M Kim HS Tong RK Bainbridge TW Vernes J-M Zhang Y. Effector-attenuating substitutions that maintain antibody stability and reduce toxicity in mice. J Biol Chem (2017) 292:3900–8. doi: 10.1074/jbc.M116.767749 PMC5339770 28077575

[251] Dickinson MJ Carlo-Stella C Morschhauser F Bachy E Corradini P Iacoboni G. Glofitamab for relapsed or refractory diffuse large B-cell lymphoma. New Engl J Med (2022) 387:2220–31. doi: 10.1056/NEJMoa2206913 36507690

[252] Pejchal R Cooper AB Brown ME Vásquez M Krauland EM. Profiling the biophysical developability properties of common igG1 fc effector silencing variants. Antibodies (2023) 12:54. doi: 10.3390/antib12030054 37753968 PMC10526015

[253] Wang X Mathieu M Brezski RJ. IgG Fc engineering to modulate antibody effector functions. Protein Cell (2018) 9:63–73. doi: 10.1007/s13238-017-0473-8 28986820 PMC5777978

[254] Wessels U Poehler A Moheysen-Zadeh M Zadak M Staack RF Umana P. Detection of antidrug antibodies against human therapeutic antibodies lacking Fc-effector functions by usage of soluble Fcγ receptor I. Bioanalysis (2016) 8:2135–45. doi: 10.4155/bio-2016-0182 27582032

[255] Newman R Hariharan K Reff M Anderson DR Braslawsky G Santoro D. Modification of the Fc region of a primatized IgG antibody to human CD4 retains its ability to modulate CD4 receptors but does not deplete CD4+ T cells in chimpanzees. Clin Immunol (2001) 98:164–74. doi: 10.1006/clim.2000.4975 11161972

[256] Bolt S Routledge E Lloyd I Chatenoud L Pope H Gorman SD. The generation of a humanized, non-mitogenic CD3 monoclonal antibody which retains in *vitro* immunosuppressive properties. Eur J Immunol (1993) 23:403–11. doi: 10.1002/eji.1830230216 8436176

[257] Jacobsen FW Stevenson R Li C Salimi-Moosavi H Liu L Wen J. Engineering an IgG scaffold lacking effector function with optimized developability. J Biol Chem (2017) 292:1865–75. doi: 10.1074/jbc.M116.748525 PMC5290958 27994062

[258] Li M Zhao R Chen J Tian W Xia C Liu X. Next generation of anti-PD-L1 atezolizumab with enhanced anti-tumor efficacy in vivo. Sci Rep (2021) 11:5774. doi: 10.1038/s41598-021-85329-9 33707569 PMC7952408

[259] Labrijn AF Janmaat ML Reichert JM Parren PWHI. Bispecific antibodies: a mechanistic review of the pipeline. Nat Rev Drug Discovery 2019 18:8 (2019) 18:585–608. doi: 10.1038/s41573-019-0028-1 31175342

[260] Chen TF Sazinsky SL Houde D DiLillo DJ Bird J Li KK. Engineering aglycosylated IgG variants with wild-type or improved binding affinity to human Fc gamma RIIA and Fc gamma RIIIAs. J Mol Biol (2017) 429:2528–41. doi: 10.1016/j.jmb.2017.07.001 PMC5584586 28694069

[261] Jo M Kwon HS Lee K-H Lee JC Jung ST. Engineered aglycosylated full-length IgG Fc variants exhibiting improved FcγRIIIa binding and tumor cell clearance. MAbs Taylor Francis (2018) pp:278–89. doi: 10.1080/19420862.2017.1402995 PMC5825196 29173039

[262] Jung ST Reddy ST Kang TH Borrok MJ Sandlie I Tucker PW. Aglycosylated IgG variants expressed in bacteria that selectively bind FcγRI potentiate tumor cell killing by monocyte-dendritic cells. Proc Natl Acad Sci (2010) 107:604–9. doi: 10.1073/pnas.0908590107 PMC2818909 20080725

[263] Ju M-S Na J-H Yu YG Kim J-Y Jeong C Jung ST. Structural consequences of aglycosylated IgG Fc variants evolved for FcγRI binding. Mol Immunol (2015) 67:350–6. doi: 10.1016/j.molimm.2015.06.020 26153451

[264] Kim JK Firan M Radu CG Kim CH Ghetie V Ward ES. Mapping the site on human IgG for binding of the MHC class I-related receptor, FcRn. Eur J Immunol (1999) 29:2819–25. doi: 10.1002/(SICI)1521-4141(199909)29:09<2819::AID-IMMU2819>3.0.CO;2-6 10508256

[265] Grevys A Nilsen J Sand KMK Daba MB Øynebråten I Bern M. A human endothelial cell-based recycling assay for screening of FcRn targeted molecules. Nat Commun (2018) 9:621–1. doi: 10.1038/s41467-018-03061-x PMC5809500 29434196

[266] Ober RJ Martinez C Vaccaro C Zhou J Ward ES. Visualizing the site and dynamics of IgG salvage by the MHC class I-related receptor, FcRn. J Immunol (2004) 172:2021–9. doi: 10.4049/jimmunol.172.4.2021 14764666

[267] Robbie GJ Criste R Dall'Acqua WF Jensen K Patel NK Losonsky GA. A novel investigational Fc-modified humanized monoclonal antibody, motavizumab-YTE, has an extended half-life in healthy adults. Antimicrobial Agents chemotherapy (2013) 57:6147–53. doi: 10.1128/AAC.01285-13 PMC3837853 24080653

[268] Brady T Cayatte C Roe TL Speer SD Ji H Machiesky L. Fc-mediated functions of nirsevimab complement direct respiratory syncytial virus neutralization but are not required for optimal prophylactic protection. Front Immunol (2023) 14. doi: 10.3389/fimmu.2023.1283120 PMC10600457 37901217

[269] van de Walle I Silence K Budding K Van de Ven L Dijkxhoorn K de Zeeuw E. ARGX-117, a therapeutic complement inhibiting antibody targeting C2. J Allergy Clin Immunol (2021) 147:1420–9. doi: 10.1016/j.jaci.2020.08.028 PMC7485568 32926878

[270] Ko S Jo M Jung ST. Recent achievements and challenges in prolonging the serum half-lives of therapeutic IgG antibodies through Fc engineering. BioDrugs (2021) 35:147–57. doi: 10.1007/s40259-021-00471-0 PMC7894971 33608823

[271] Borghi S Bournazos S Thulin NK Li C Gajewski A Sherwood RW. FcRn, but not FcγRs, drives maternal-fetal transplacental transport of human IgG antibodies. Proc Natl Acad Sci (2020) 117:12943–51. doi: 10.1073/pnas.2004325117 PMC7293622 32461366

[272] Kulasekararaj AG Hill A Rottinghaus ST Langemeijer S Wells R Gonzalez-Fernandez FA. Ravulizumab (ALXN1210) vs eculizumab in C5-inhibitor-experienced adult patients with PNH: the 302 study. Blood (2019) 133:540–9. doi: 10.1182/blood-2018-09-876805 PMC6368201 30510079

[273] Vu T Ortiz S Katsuno M Annane D Mantegazza R Beasley KN. Ravulizumab pharmacokinetics and pharmacodynamics in patients with generalized myasthenia gravis. J Neurol (2023) 270:3129–37. doi: 10.1007/s00415-023-11617-1 PMC10188401 36890354

[274] Levin MJ Ustianowski A De Wit S Launay O Avila M Templeton A. Intramuscular AZD7442 (tixagevimab–cilgavimab) for prevention of COVID-19. New Engl J Med (2022) 386:2188–200. doi: 10.1056/NEJMoa2116620 PMC9069994 35443106

[275] Evering TH Chew KW Giganti MJ Moser C Pinilla M Wohl DA. Safety and efficacy of combination SARS-coV-2 neutralizing monoclonal antibodies amubarvimab plus romlusevimab in nonhospitalized patients with COVID-19. Ann Internal Med (2023) 176:658–66. doi: 10.7326/M22-3428 PMC10150320 37068272

[276] Hinton PR Johlfs MG Xiong JM Hanestad K Ong KC Bullock C. Engineered human IgG antibodies with longer serum half-lives in primates. J Biol Chem (2004) 279:6213–6. doi: 10.1074/jbc.C300470200 14699147

[277] Datta-Mannan A Chow C-K Dickinson C Driver D Lu J Witcher DR. FcRn affinity-pharmacokinetic relationship of five human IgG4 antibodies engineered for improved in *vitro* FcRn binding properties in cynomolgus monkeys. Drug Metab Disposition (2012) 40:1545–55. doi: 10.1124/dmd.112.045864 22584253

[278] Vaccaro C Zhou J Ober RJ Ward ES. Engineering the Fc region of immunoglobulin G to modulate in *vivo* antibody levels. Nat Biotechnol (2005) 23:1283–8. doi: 10.1038/nbt1143 16186811

[279] Ward ES Ober RJ. Targeting FcRn to generate antibody-based therapeutics. Trends Pharmacol Sci (2018) 39:892–904. doi: 10.1016/j.tips.2018.07.007 30143244 PMC6169532

[280] Howard JF Jr. Bril V Vu T Karam C Peric S Margania T. Safety, efficacy, and tolerability of efgartigimod in patients with generalised myasthenia gravis (ADAPT): a multicentre, randomised, placebo-controlled, phase 3 trial. Lancet Neurol (2021) 20:526–36. doi: 10.1016/S1474-4422(21)00159-9 34146511

[281] Ulrichts P Guglietta A Dreier T van Bragt T Hanssens V Hofman E. Neonatal Fc receptor antagonist efgartigimod safely and sustainably reduces IgGs in humans. J Clin Invest (2018) 128(10):4372–86. doi: 10.1172/JCI97911 PMC6159959 30040076

[282] Goebeler M Bata-Csörgő Z De Simone C Didona B Remenyik E Reznichenko N. Treatment of pemphigus vulgaris and foliaceus with efgartigimod, a neonatal Fc receptor inhibitor: a phase II multicentre, open-label feasibility trial. Br J Dermatol (2022) 186(3):429–39. doi: 10.1111/bjd.20782 34608631

[283] Igawa T Maeda A Haraya K Tachibana T Iwayanagi Y Mimoto F. Engineered monoclonal antibody with novel antigen-sweeping activity in vivo. PloS One (2013) 8:e63236–6. doi: 10.1371/journal.pone.0063236 PMC3646756 23667591

[284] Yang D Giragossian C Castellano S Lasaro M Xiao H Saraf H. Maximizing in *vivo* target clearance by design of pH-dependent target binding antibodies with altered affinity to FcRn. MAbs Taylor Francis (2017), 1105–17. doi: 10.1080/19420862.2017.1359455 PMC5627591 28786732

[285] Leabman MK Meng YG Kelley RF DeForge LE Cowan KJ Iyer S. Effects of altered FcγR binding on antibody pharmacokinetics in cynomolgus monkeys. MAbs Taylor Francis (2013) 5(6):896–903. doi: 10.4161/mabs.26436 PMC3896603 24492343

[286] Einarsdottir HK Selman MHJ Kapur R Scherjon S Koeleman CAM Deelder AM. Comparison of the Fc glycosylation of fetal and maternal immunoglobulin G. Glycoconjugate J (2013) 30(2):147–57. doi: 10.1007/s10719-012-9381-6 PMC3552368 22572841

[287] Datta-Mannan A Thangaraju A Leung D Tang Y Witcher DR Lu J. Balancing charge in the complementarity-determining regions of humanized mAbs without affecting pI reduces non-specific binding and improves the pharmacokinetics. MAbs Taylor Francis (2015) 7(3):483–93. doi: 10.1080/19420862.2015.1016696 PMC4622971 25695748

[288] Piche-Nicholas NM Avery LB King AC Kavosi M Wang M O'Hara DM. Changes in complementarity-determining regions significantly alter IgG binding to the neonatal Fc receptor (FcRn) and pharmacokinetics. MAbs Taylor Francis (2018), 81–94. doi: 10.1080/19420862.2017.1389355 PMC5800364 28991504

[289] Gjølberg TT Frick R Mester S Foss S Grevys A Høydahl LS. Biophysical differences in IgG1 Fc-based therapeutics relate to their cellular handling, interaction with FcRn and plasma half-life. Commun Biol (2022) 5:832–2. doi: 10.1038/s42003-022-03787-x PMC9388496 35982144

[290] Grevys A Frick R Mester S Flem-Karlsen K Nilsen J Foss S. Antibody variable sequences have a pronounced effect on cellular transport and plasma half-life. Iscience (2022) 25:103746–6. doi: 10.1016/j.isci.2022.103746 PMC8800109 35118359

[291] Grevys A Bern M Foss S Bratlie DB Moen A Gunnarsen KS. Fc engineering of human IgG1 for altered binding to the neonatal Fc receptor affects Fc effector functions. J Immunol (2015) 194(11):5497–508. doi: 10.4049/jimmunol.1401218 PMC4432726 25904551

[292] Kaplon H Crescioli S Chenoweth A Visweswaraiah J Reichert JM. Antibodies to watch in 2023. Mabs Taylor Francis (2023) 15(1):2153410–2153410. doi: 10.1080/19420862.2022.2153410 PMC9728470 36472472

[293] Saunders KO. Conceptual approaches to modulating antibody effector functions and circulation half-life. Front Immunol (2019) 10:1296–6. doi: 10.3389/fimmu.2019.01296 PMC6568213 31231397

[294] Ishii T Ishida T Utsunomiya A Inagaki A Yano H Komatsu H. Defucosylated humanized anti-CCR4 monoclonal antibody KW-0761 as a novel immunotherapeutic agent for adult T-cell leukemia/lymphoma. Clin Cancer Res (2010) 16:1520–31. doi: 10.1158/1078-0432.CCR-09-2697 20160057

[295] Kolbeck R Kozhich A Koike M Peng L Andersson CK Damschroder MM. MEDI-563, a humanized anti–IL-5 receptor α mAb with enhanced antibody-dependent cell-mediated cytotoxicity function. J Allergy Clin Immunol (2010) 125:1344–1353.e2. doi: 10.1016/j.jaci.2010.04.004 20513525

[296] Bartelds GM de Groot E Nurmohamed MT Hart MHL van Eede PH Wijbrandts CA. Surprising negative association between IgG1 allotype disparity and anti-adalimumab formation: a cohort study. Arthritis Res Ther (2010) 12:1–7. doi: 10.1186/ar3208 PMC3046534 21187010

[297] Webster CI Bryson CJ Cloake EA Jones TD Austin MJ Karle AC. A comparison of the ability of the human IgG1 allotypes G1m3 and G1m1,17 to stimulate T-cell responses from allotype matched and mismatched donors. mAbs (2016) 8:253–63. doi: 10.1080/19420862.2015.1128605 PMC4966604 26821574

[298] Dumet C Pottier J Gouilleux-Gruart V Watier H. Insights into the IgG heavy chain engineering patent landscape as applied to IgG4 antibody development. MAbs Taylor Francis (2019) 11(8):1341–50. doi: 10.1080/19420862.2019.1664365 PMC6816381 31556789

[299] Suurs FV Lub-de Hooge MN de Vries EGE de Groot DJA. A review of bispecific antibodies and antibody constructs in oncology and clinical challenges. Pharmacol Ther (2019) 201:103–19. doi: 10.1016/j.pharmthera.2019.04.006 31028837

[300] Strop P Ho WH Boustany LM Abdiche YN Lindquist KC Farias SE. Generating bispecific human IgG1 and IgG2 antibodies from any antibody pair. J Mol Biol (2012) 420:204–19. doi: 10.1016/j.jmb.2012.04.020 22543237

[301] Labrijn AF Meesters JI de Goeij BECG van den Bremer ETJ Neijssen J Kampen MDv. Efficient generation of stable bispecific IgG1 by controlled Fab-arm exchange. Proc Natl Acad Sci (2013) 110:5145–50. doi: 10.1073/pnas.1220145110 PMC3612680 23479652

[302] Labrijn AF Meesters JI Priem P De Jong RN Van Den Bremer ETJ Van Kampen MD. Controlled Fab-arm exchange for the generation of stable bispecific IgG1. Nat Protoc (2014) 9:2450–63. doi: 10.1038/nprot.2014.169 25255089

[303] Namisaki H Saito S Hiraishi K Haba T Tanaka Y Yoshida H. R409K mutation prevents acid-induced aggregation of human IgG4. PloS One (2020) 15:e0229027–e0229027. doi: 10.1371/journal.pone.0229027 32182240 PMC7077836

[304] Borrok MJ Wu Y Beyaz N Yu X-Q Oganesyan V Dall'Acqua WF. pH-dependent binding engineering reveals an FcRn affinity threshold that governs IgG recycling. J Biol Chem (2015) 290:4282–90. doi: 10.1074/jbc.M114.603712 PMC4326836 25538249

[305] Escobar-Cabrera E Lario P Baardsnes J Schrag J Durocher Y Dixit S. Asymmetric fc engineering for bispecific antibodies with reduced effector function. Antibodies (2017) 6(2):7. doi: 10.3390/antib6020007 31548523 PMC6698841

[306] Chen D Zhao Y Li M Shang H Li N Li F. A general Fc engineering platform for the next generation of antibody therapeutics. Theranostics (2021) 11:1901–1. doi: 10.7150/thno.51299 PMC7778609 33408788

[307] van Tilburg SJ Jacobs BC Ooijevaar-de Heer P Fokkink WJR Huizinga R Vidarsson G. Novel approach to monitor intravenous immunoglobulin pharmacokinetics in humans using polymorphic determinants in IgG1 constant domains. Eur J Immunol (2022) 52:609–17. doi: 10.1002/eji.202149653 34854474

